# Biological Control Agents Against Fusarium Wilt of Banana

**DOI:** 10.3389/fmicb.2019.00616

**Published:** 2019-04-05

**Authors:** Giovanni Bubici, Manoj Kaushal, Maria Isabella Prigigallo, Carmen Gómez-Lama Cabanás, Jesús Mercado-Blanco

**Affiliations:** ^1^Consiglio Nazionale delle Ricerche (CNR), Istituto per la Protezione Sostenibile delle Piante (IPSP), Bari, Italy; ^2^International Institute of Tropical Agriculture (IITA), Dar es Salaam, Tanzania; ^3^Department of Crop Protection, Institute for Sustainable Agriculture (CSIC), Córdoba, Spain

**Keywords:** *Musa acuminata*, *Fusarium oxysporum* f. sp. *cubense*, Panama disease, soil microbiota, beneficial microorganisms, biocontrol

## Abstract

In the last century, the banana crop and industry experienced dramatic losses due to an epidemic of Fusarium wilt of banana (FWB), caused by *Fusarium oxysporum* f.sp. *cubense* (*Foc*) race 1. An even more dramatic menace is now feared due to the spread of *Foc* tropical race 4. Plant genetic resistance is generally considered as the most plausible strategy for controlling effectively such a devastating disease, as occurred for the first round of FWB epidemic. Nevertheless, with at least 182 articles published since 1970, biological control represents a large body of knowledge on FWB. Remarkably, many studies deal with biological control agents (BCAs) that reached the field-testing stage and even refer to high effectiveness. Some selected BCAs have been repeatedly assayed in independent trials, suggesting their promising value. Overall under field conditions, FWB has been controlled up to 79% by using *Pseudomonas* spp. strains, and up to 70% by several endophytes and *Trichoderma* spp. strains. Lower biocontrol efficacy (42–55%) has been obtained with arbuscular mycorrhizal fungi, *Bacillus* spp., and non-pathogenic *Fusarium* strains. Studies on *Streptomyces* spp. have been mostly limited to *in vitro* conditions so far, with very few pot-experiments, and none conducted in the field. The BCAs have been applied with diverse procedures (e.g., spore suspension, organic amendments, bioformulations, etc.) and at different stages of plant development (i.e., *in vitro*, nursery, at transplanting, post-transplanting), but there has been no evidence for a protocol better than another. Nonetheless, new bioformulation technologies (e.g., nanotechnology, formulation of microbial consortia and/or their metabolites, etc.) and tailor-made consortia of microbial strains should be encouraged. In conclusion, the literature offers many examples of promising BCAs, suggesting that biocontrol can greatly contribute to limit the damage caused by FWB. More efforts should be done to further validate the currently available outcomes, to deepen the knowledge on the most valuable BCAs, and to improve their efficacy by setting up effective formulations, application protocols, and integrated strategies.

## Introduction

**Figure 1:**
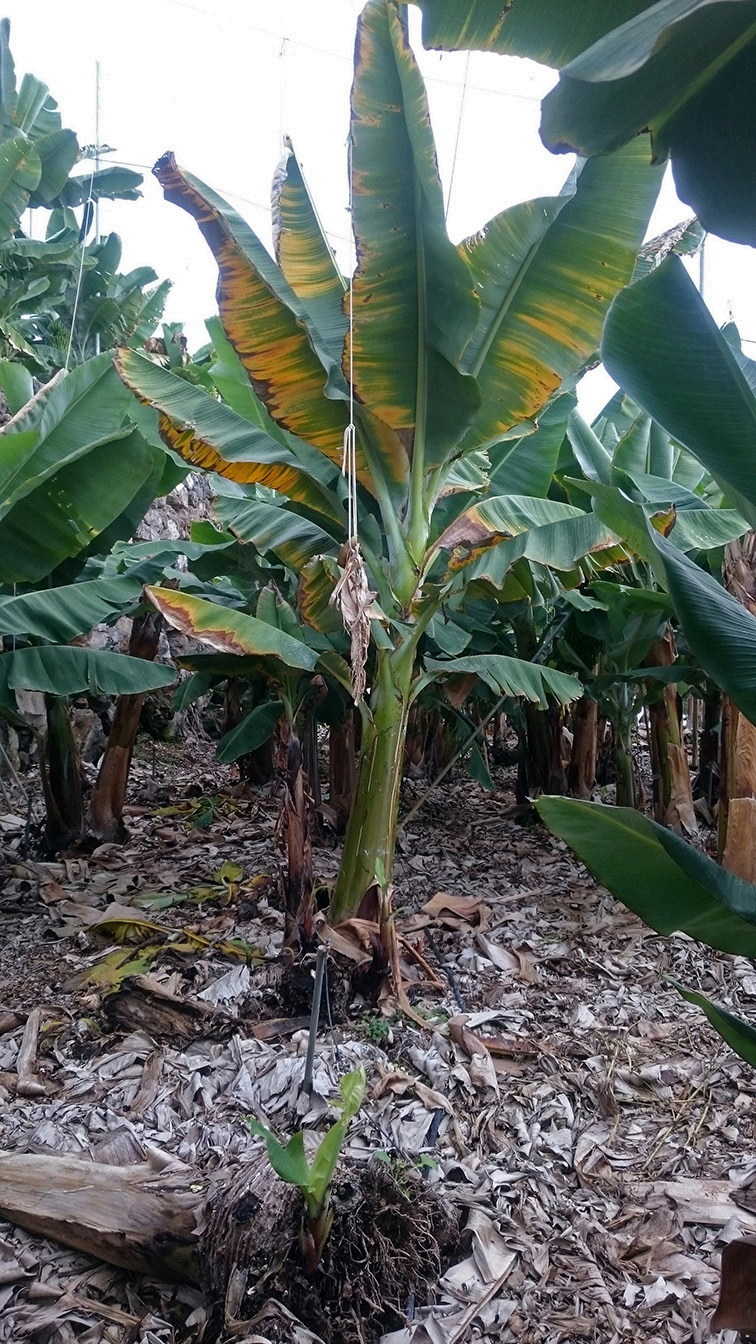
Typical symptoms of Fusarium wilt on a banana plant cv. Pequeña Enana (or “Dwarf Cavendish”; AAA genome) in Tenerife.

Among plant pathologists, everybody knows the story of Fusarium wilt of banana (FWB), also known as Panama disease. Until the 1950s, the cultivar Gros Michel had dominated the panorama of cultivated banana worldwide. The global banana production was threatened by a destructive soil-borne fungus, namely *Fusarium oxysporum* f. sp. *cubense* (*Foc*) (Figure 1). Fortunately, a resistant cultivar was identified, the “Cavendish.” Hence, due to its resistance to the races 1 and 2 of *Foc* (*Foc* R1 and R2), “Cavendish” was used to replace universally the “Gros Michel,” which unique fruit flavor has become virtually a distant memory. The global banana industry was saved until the 1990s, when a *Foc* strain virulent on “Cavendish” emerged, the race 4 (R4). First identified in Taiwan, *Foc* race 4 (R4) rapidly spread to South East Asian countries (e.g., Indonesia, Malaysia, and the Philippines), China, northern Australia, India, Pakistan, Middle East countries (e.g., Jordan, Israel, and Lebanon) and Africa (Mozambique) (Vézina, 2018a). *Foc* R4 strains are further separated into tropical race 4 (TR4) and subtropical race 4 (STR4), based on the evidence that the latter group necessitates predisposing factors, such as low temperatures, to cause the disease. The race 4 affects not only “Cavendish,” but also R1- and R2-susceptible varieties, while R1 affects “Gros Michel,” “Silk” and “Pisang Awak,” and R2 affects “Bluggoe” (Ploetz and Pegg, 2000). The race 3 (R3), which infects *Heliconia* spp., is no longer included in the *Foc* species (Ploetz, 2005). Currently, *Foc* TR4 is present in 19 of the 135 countries producing bananas (Dusunceli, 2017; Zheng et al., 2018), and its alarming spread has gained a remarkable interest from world media and cultural community (Butler, 2013; Gittleson, 2018).

Nowadays, bananas (including plantains and other cooking bananas) are the most produced fruit on the Earth (148 million tons produced in 2016 in 135 countries) and provide a staple for some 400 million people worldwide (Dusunceli, 2017). Its production and cultivated lands have progressively increased over the years (FAO, 2018b). Under the climate change scenario, the temperature increase will make conditions more favorable for banana production in the subtropical and tropical highlands. Land area suitable for bananas is estimated to augment 50% by 2070 (FAO, 2016). Currently, Cavendish varieties cover ca. 40% of the global production (the export trade accounts for 15% of the global production), and may be well the only bananas present on supermarket shelves of non-producer countries, because they dominate the export trade for a 12 billion dollars affair (FruiTrop, 2018). Therefore, it is clear that the economic impact of a second *Foc* epidemic would be more dramatic than the first one. As demonstrated in the past for the *Foc* R1 plague, the host genetic resistance is the better way to tackle the virulent strain. So far, however, no commercial varieties displaying an effective resistance against *Foc* R4 are available. Several research projects (Kema, 2018) and international initiatives, such as Promusa (Van den Bergh et al., 2018), World Banana Forum (Liu and Prada, 2018), the African Consortium for TR4 (Viljoen et al., 2018), and the four banana regional research networks MUSALAC, Innovate Plantain, BARNESA, and BAPNET (Bioversity International, 2018) have been started with the aim to save this crop. Lastly, the research project “Microbial Uptakes for Sustainable Management of Major Banana Pests and Diseases (MUSA)” (Horizon 2020 framework), in which we are involved, aims at improving the sustainable protection of banana crops from three major biotic constraints: FWB, nematodes, and banana weevil. The MUSA project holistically encompasses integrated pest management methods based on microbial consortia and available banana and enset germplasms, including newly developed elite hybrids (Ciancio, 2017).

Biological control has gained great interest in the last years in many pathosystems, including *Foc*/banana. This has been mainly due to the large input of pesticides, which cause economic, environmental and safety concerns. Biological control must not be a strategy limited to organic farming (adopted in 1% of banana cultivated lands) (Liu and Prada, 2018), but included within integrated disease management frameworks implemented in agricultural systems. Historically, biological control has suffered from inconsistent results over the seasons and the environments, most likely due to interacting variables (e.g., environmental, genetic, physiological, etc.) present in any given agro-ecosystem, and that are not fully understood or difficult to control. Factors affecting the biocontrol efficacy of FWB have been summarized by Guo et al. (2013).

In the present article, we report the impact of FWB in different continents and the overall disease management strategies with special regard to biological control. Thus, we report a comprehensive literature review of the biological control agents (BCAs) used against *Foc*, we also analyze critically the most relevant results achieved, and identify gaps in our current knowledge of this control strategy in order to (i) show the actual potential of biological control of FWB, and (ii) foster new research lines based on currently-available powerful technologies that will aid in developing novel and more effective biocontrol tools.

## Disease Impact on Banana Production …

### … in Asia and Australia

Since 1967, the pathogen has caused severe damage in Taiwan, the major exporter of banana to Japan till that time, and the lands cultivated with banana have passed from 50,000 ha in the 1960s to about 6,000 ha in the 2000s. Banana plantations have been also decimated in Indonesia and Malaysia in the early 1990s. The spread of *Foc* TR4 has aggravated the condition in Asia (Molina et al., 2009). In the Philippines, *Foc* has been present since 1970, but TR4 has been detected in 2006. Then, hectares of banana plantations have been abandoned by farmers because of *Foc* TR4, resulting in an annual loss of 3 billion dollars, and approximately 66,000 families lost their livelihood (Molina et al., 2009). As a consequence, in 2014 the Federation of Cooperatives in Mindanao (FEDCO) advised banana growers to shift to oil palm in fields compromised by *Foc*, a crop suitable for the Philippines lands and with increasing market demand (Carillo, 2014). During a survey in 2006, about 6,700 ha of banana plantations were found severely affected by the pathogen in the Guangdong province, southern China (Yi et al., 2007), and in 2012 an extensive damage was also observed around the Guangxi's capital and the Hainan island (Farquhar, 2012). India is the largest producer of bananas in the world with 28.6 million tons produced annually (FAO, 2018b), 70% of which is of the Cavendish cultivar Grand Naine (AAA genome group). There, six vegetative compatibility groups (VCGs) of *Foc* have been found, and disease severity has been as high as 80–90% on susceptible cultivars such as “Silk” (AAB), “Ney Poovan” (AB), “Pisang Awak” (ABB), “Pome” (AAB), “Bluggoe” (ABB), “Monthan” (ABB), and “Mysore” (AAB) (the latter was resistant to *Foc*, but then found infected by VCG 0124/0125) (Mustaffa and Thangavelu, 2011). *Foc* TR4 has been first found in 2015 in the state of Bihar, in the northeastern part of the country, and then detected in the states of Uttar Pradesh, Madhya Pradesh, and Gujarat, on the west coast. It has been officially confirmed only in 2017 (Damodaran et al., 2018). It has been estimated that *Foc* TR4 can inflict losses for 500 billion Indian rupees (ca. 7 billion American dollars) to the country's banana industry (Kulkarni, 2018).

The most recent records of TR4 have come from Jordan, Lebanon, Pakistan, Laos, Vietnam and Myanmar (Molina et al., 2009; Zheng et al., 2018). In 2016, two TR4 outbreak areas were identified in Israel, where the affected farms were promptly fenced in, and diseased plants destroyed. In May 2018, the Israel's National Plant Protection Organization officially declared the TR4 eradicated from Israel (EPPO Reporting Service, 2018). Although the public declaration, scientists know that *Foc* cannot be eradicated from the soil.

FWB in Australia has been reviewed previously by Pegg et al. (1996). Australia was the continent where *Foc* was reported for the first time (Bancroft, 1876), whereas *Foc* TR4 has been reported in the Northern Territory since 1997 (Bentley et al., 2001; Conde and Pitkethley, 2001). Later, in Queensland, three TR4 spots were identified between 2015 and 2018. In Australia, the severe biosecurity regulations have been very effective, and have made the *Foc* TR4 spread extremely slower than elsewhere. In September 2018, Biosecurity Queensland in partnership with Biosecurity Solutions Australia has announced the development of a certification system for TR4-infested farms that meet the requirements for severe food safety and biosecurity standards (Northern Queensland Register, 2018). The lack of resistant cultivar has drastically reduced the banana production in Australia, with a commercial industry loss above 90% and an augmented price of bananas in domestic markets (Cook et al., 2015).

### … in Latin America and the Caribbean

Latin America and the Caribbean (LAC) support 28% of the global banana production and provide more than 80% of the banana exports (FAO, 2018b). According to Dita et al. (2013), FWB in LAC impact on certain production systems more than others. Particularly affected systems are the monocultures such as “Prata” (AAB) in Brazil, “Isla” and “Palillo” (AAB, Pacific plantain) in Peru, and the banana plantations intercropped with coffee and cocoa. Due to the *Foc* R1 epidemic, not only “Gros Michel” (AAA) was replaced with “Cavendish” (AAA), but also “Prata Ana” (AAB) (in Brazil) and apple banana (AAB). The cooking banana “Bluggoe” (ABB) was replaced with “Pelipita” (ABB), which is also resistant to Moko (*Ralstonia solanacearum* race 2). With the “Cavendish” advent, FWB disappeared as a problem for the trades (Buddenhagen, 1990), while the black leaf streak (or black Sigatoka, caused by *Mycosphaerella fijiensis*) assumed the primary importance (Ploetz, 2015a). Then, the rapid spread of *Foc* TR4 has recovered the importance of FWB, though black leaf streak remains of high economic relevance, especially in tropical regions. At present, the banana industry in LAC is almost totally based on Cavendish varieties, and the limited production for national markets is based on apple banana, plantain and “Gros Michel.” In countries of LAC, *Foc* TR4 is not present, therefore the pathogen exclusion by means of strong quarantine procedures is compulsory. Farmers, technicians, and politicians of LAC became sensitized of the FWB menace from the past, and they continue to become aware thanks to dissemination and technical events, workshops and meetings (Clercx, 2013; FAO, 2018c). An awareness campaign has been launched by a consortium of institutes in order to emphasize the importance of quarantine measures in preventing the entrance of *Foc* TR4 into LAC (Pocasangre et al., 2011).

### … in Africa

Banana has been present on the continent for over 1400 years (Blomme et al., 2013). It is an important staple food for African people, as in countries such as Uganda, Rwanda, and Cameroon per capita consumption exceeds well 200 kg of bananas, so providing up to 25–35% of the daily nutrient intake. About 70–80% of banana production is consumed locally (FAO, 2018a; Viljoen et al., 2018).

*Foc* race 1 has first been reported from West Africa in 1924, and then from Tanzania in 1951 (Blomme et al., 2013; Viljoen et al., 2018). Vegetative compatibility groups and phylogenies of *Foc* populations in Africa have been extensively studied (reviewed by Blomme et al., 2013). Until the year 2000, FWB was reported in two out of the six production areas of South Africa: Kiepersol and southern KwaZulu-Natal. In KwaZulu-Natal, the disease appeared in 1940 and spread to Kiepersol with infected plant material, where it resulted in a 30% loss of banana plantations during the 1990s. At that time, only *Foc* STR4 occurred in South Africa (Viljoen, 2002). In Uganda, the major producer of cooking bananas worldwide with 3.7 million tons in 2016 (FAO, 2018b), FWB is less relevant than weevil [*Cosmopolites sordidus* (Germar)] and Xanthomonas wilt (*Xanthomonas campestris* pv. *musacearum*) and is present at altitudes above 1,300 m (Vézina, 2018b). With the occurrence in one farm in northern Mozambique, *Foc* TR4 officially entered the African continent in 2013, although it was likely an earlier presence (Butler, 2013). There, *Foc* TR4 destroyed about one million plants at a rate of approximately 15,000 plants per week (Jansen, 2017). After the detection of *Foc* TR4 in the six farms of Metochéria (about 1,500 ha cultivated with banana), the access of people and the movement of farm personnel and international staff have been restricted and controlled in order to avoid further spread of the pathogen (Viljoen et al., 2018).

The “Cavendish” varieties are not widely grown in Africa (about 10% of the region's production), whereas plantains (AAB) predominate in West Africa (71% of the production) with about 100 varieties, and highland banana (AAA; East Africa Highland Banana or EAHB) and other cooking types (ABB) predominate in East Africa (71% of production) with more than 50 varieties (FruiTrop, 2018; Viljoen et al., 2018). “Cavendish” was used in countries like Kenya to replace the susceptible varieties “Kampala” (“Gros Michel”) and “Bokoboto” (“Bluggoe,” ABB), susceptible to *Foc* R1. Besides these exceptions and few others (Sebasigari and Stover, 1988; Rutherford, 2001), African germplasm is largely resistant to *Foc* R1 and R2, but its reaction to *Foc* TR4 is still unknown. This scenario would make the *Foc* TR4 diffusion subtler than in a “Cavendish” monoculture, since new TR4 spots could be undetected properly or assumed to be caused by already established races but not TR4 (Vézina, 2018c). In addition, the fact that sucker-derived plant material is still used more than tissue culture plants in Africa (Dubois et al., 2013; Niere et al., 2014) may contribute to exacerbating the problem.

### … in the Canary Islands

Banana is the most important intensive agricultural crop in the Canary Islands, an archipelago located in the Atlantic Ocean between 27° 37′-29° 25′ N and 13° 20′-18° 10′ W. The orography and pedological conditions have conditioned banana cultivation in these islands, shaping a particular landscape of terraces built up over volcanic stones and debris. Banana farms are usually small and about 80% of them have <1 hectare (Instituto Canario de Calidad Agroalimentaria, 2018). To check historical and current figures on banana production and cultivated area per island and municipalities, interested readers can consult the Canary Islands government's website (Gobierno de Canarias, 2018). Cultivar “Pequeña Enana” (or “Dwarf Cavendish,” AAA) is overwhelmingly predominant in the islands, with a minor and more recent presence of “Gran Enana” (or “Grande Naine” or “Grand Nain”; AAA) as well as local selections from “Pequeña Enana” like “Gruesa,” “Brier,” and “Negrita” (Azkolain Olaondo, 2016). Banana production in the archipelago is highly technified with practices such as artificial soil preparation, use of *in vitro* propagated plants from selected cultivars, modern fertigation approaches and extended use of greenhouse cultivation systems (Azkolain Olaondo, 2016). In Tenerife, Fusarium wilt was detected for the first time in the 1920s (Blomme et al., 2013). It is currently present in any area of the archipelago where banana is cultivated, showing an incidence of affected plants ranging from 2 to 12%. In some cases, however, incidence has been reported to be much higher causing more than 30% of crop loss in specific spots (Rodríguez Serrano, 2012). Studies carried out to determine the racial structure and pathogenicity of *Foc* isolates obtained from infected plants and soils of “Dwarf Cavendish” and “Grande Naine” plantations pointed to the fact that there are no differences among pathogen populations (Regalado Guijarro and Hernández Hernández, 1998). The presence and virulence of *Foc* STR4 have been well determined in Tenerife, and this race has also been claimed to be responsible for Dwarf Cavendish infections occurring in Gran Canaria island (Ploetz et al., 1990; Domínguez-Hernández et al., 2008).

## Overall Control Strategies Currently Adopted

Available and currently-implemented measures to control FWB have been recently compiled (Ploetz, 2015b; Dita et al., 2018; Siamak and Zheng, 2018), and comprehensive information are also available in web portals such as World Banana Forum (Clercx, 2013) and Promusa (Van den Bergh et al., 2018). Previously, other authors have also produced similar reviews (e.g., Figueroa, 1987; Jeger et al., 1996; Wui, 2000; Murray, 2001; Pocasangre et al., 2011; Pérez-Vicente, 2015).

*Foc* is particularly difficult to control for a number of reasons: (a) it is a soil-borne fungus with a long survival in the soil (more than 20 years), even in the absence of plant hosts (Stover, 1962; Buddenhagen, 2009), or within alternate hosts which do not necessarily show disease symptoms (Waite and Stover, 1960; Pittaway et al., 1999; Hennessy et al., 2005); (b) being a vascular pathogen, it escapes the contact with the control means (e.g., non-systemic fungicides, non-endophytic BCAs, etc.) once it penetrates into the plant; (c) it can be spread by banana vegetative propagation material, soil vectored by workers and machinery, irrigation water, etc.; and (d) the banana monoculture, especially Cavendish varieties in the case of *Foc* TR4, facilitates the pathogen spread.

Overall, fungicides have provided unsatisfactory control levels. *In vitro*, toxicity against *Foc* has been proved for phosphonate, ambuic acid, organotin mandelates, carbendazim, carboxin, propiconazole, benomyl, and difenoconazole (Davis et al., 1994; Davis and Grant, 1996; Li et al., 2001; Araujo et al., 2004; Somu et al., 2014). *In planta*, only a few research articles have reported a significant disease control by using fungicides (e.g., carbendazim) (Lakshmanan and Selvaraj, 1984; Eswaramurthy et al., 1988; Roy et al., 1998) or resistance inducers (e.g., indoleacetic acid and menadione sodium bisulphite) (Fernández-Falcón et al., 2003; Borges et al., 2004). Until now, however, these pot-experiments have not been validated under field conditions.

In the lack of highly effective control means, like available sources of host genetic resistance, plant diseases are usually managed by integrated frameworks, with an emphasis in preventive measures. This is particularly true for soil-borne diseases like FWB and Verticillium wilts, whose causal pathogens cannot be eradicated once contaminate the soil. With few context-specific differences, integrated disease management strategies of diverse vascular diseases have much in common (Bubici and Cirulli, 2008; Cirulli et al., 2010; López Escudero and Mercado-Blanco, 2011; Jiménez-Díaz et al., 2012).

**Figure 2:**
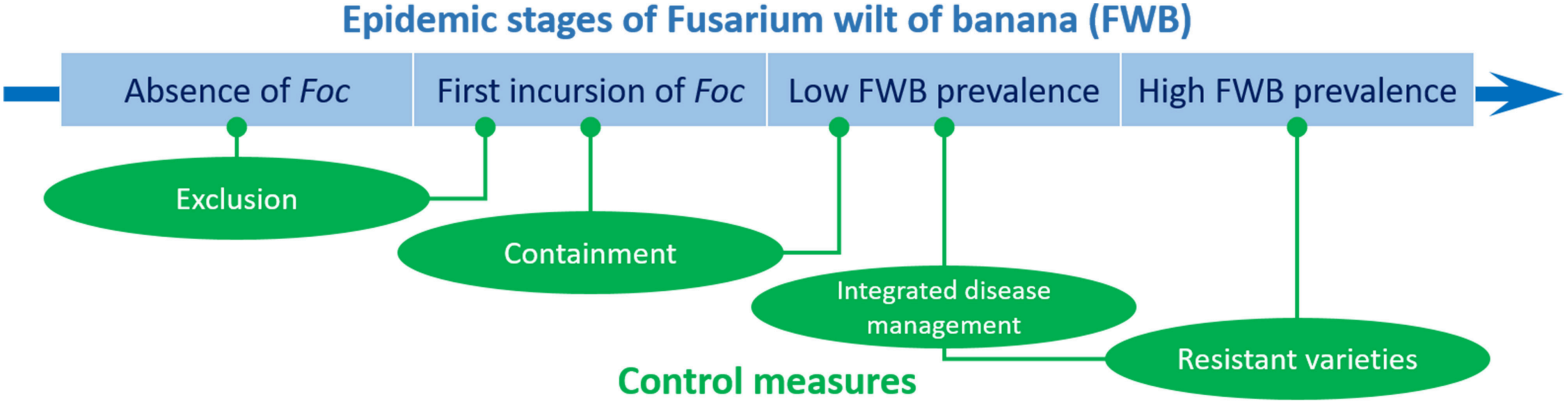
Control strategies of Fusarium wilt of banana at different epidemic stages. In the absence of *Fusarium oxysporum* f. sp. *cubense* (*Foc*), exclusion measures must be used to prevent the pathogen entrance. At the first incursion of *Foc*, containment measures must be rapidly initiated. Once *Foc* is established, containment measures must be continued, and integrated disease management can be adopted. With a high disease prevalence, the use of resistant varieties is the only way to successfully combat FWB.

In the case of FWB, Dita et al. (2018) stressed that the implementation and integration of some disease management practices may vary according to four main farm-level scenarios, which indeed correspond to four stages of the disease epidemic: (i) absence of *Foc*, (ii) first incursion of *Foc*, (iii) low FWB prevalence, and (iv) high FWB prevalence (Figure 2). In the absence of a devastating pathogen like *Foc*, it is imperative to adopt exclusion measures to prevent the pathogen entrance, both at farm level using proper practices adopted by the personnel, and at the regional or national level using legal initiatives such as quarantine, certification, etc. This is the case of *Foc* TR4 in LAC and other countries where this race is not yet present. At the first incursion of *Foc*, exclusion methods still have great importance, but containment measures must be rapidly initiated and scrupulously applied. Farms in countries where *Foc* TR4 has been detected recently would be in this second situation (e.g., Mozambique, Lebanon, Pakistan, Israel, Laos, Vietnam, and Myanmar). Once *Foc* is established, exclusion tactics make no longer sense, but containment measures must be implemented, and integrated disease management can be adopted under low disease pressure. With high disease prevalence, containment measures are obviously not effective, and integrated disease management may be questionable.

Thus, the use of resistant varieties is the only way for successfully confronting FWB. As mentioned above, this was already experienced for the *Foc* R1 epidemic. On the other hand, Dita et al. (2018) have reported two examples where the integrated disease management against *Foc* R1 allows some farmers to grow the susceptible varieties “Prata”-type, in Brazil, and “Gros Michel,” in Colombia.

**Figure 3:**
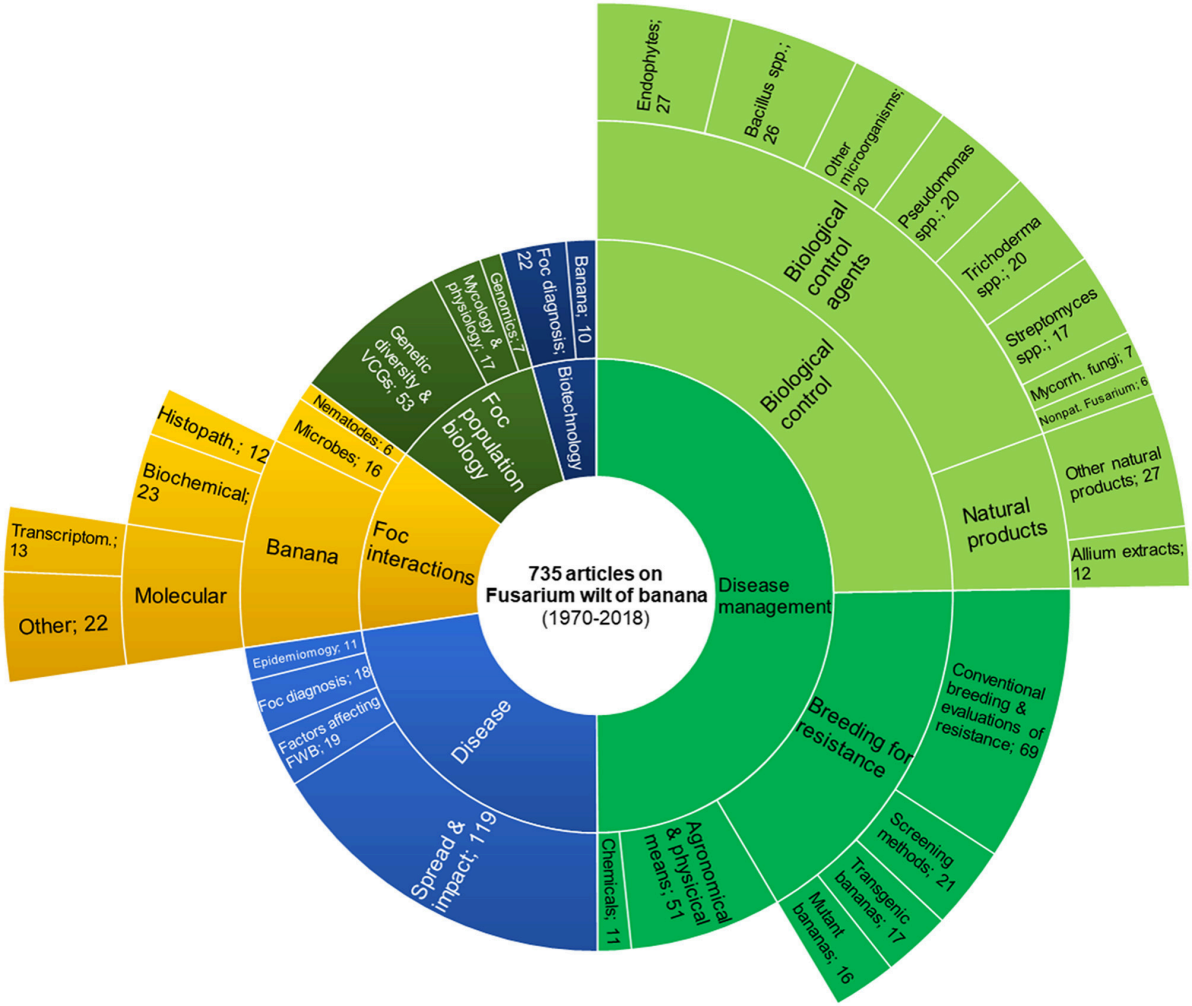
Main topics of scientific articles dealing with Fusarium wilt of banana. Articles were retrieved from the CAB Direct database (1970–2018) by searching the keywords “*Fusarium cubense*” or “Panama disease” in the title and abstract. *Foc*: *Fusarium oxysporum* f. sp. *cubense*; FWB: Fusarium wilt of banana; VCGs: vegetative compatibility groups.

Unfortunately, no commercial varieties displaying high resistance to *Foc* TR4 coupled with good agronomic traits and fruit characteristics are yet available. However, the research on breeding for resistance has been particularly active and, besides conventional breeding and screening of genotypes, several mutant, and transgenic bananas have been developed (Figure 3). Resistance sources to *Foc* TR4 have been found in banana wild relatives, especially *M. basjoo* and *M. itinerans*, but also in *M. yunnanensis, M. nagensium, M. ruiliensis*, and *M. velutina*, whereas higher disease intensity was observed in *M. balbisiana* and *M. acuminata* subsp. *burmannica* (Li et al., 2015). Within *M. acuminata*, the genotype DH-Pahang, which genome was sequenced, has been found resistant to *Foc* TR4 (D'Hont et al., 2012; Zhang et al., 2018). Other promising varieties resistant to *Foc* TR4 are being tested under field conditions. Some of them were developed by the Fundación Hondureña de Investigación Agrícola (FHIA), and others by the Taiwan Banana Research Institute (TBRI). Other resistant genotypes have been identified within the world's largest collection of bananas, owned by the International *Musa* Germplasm Transit Centre, which is managed by Bioversity International and hosted by the Katholieke Universiteit Leuven in Belgium (Vézina, 2018a). The so-called “Giant Cavendish” tissue-culture variants (GCTCV) have been selected for the resistance against *Foc* TR4 by the Taiwan Banana Research Institute (TBRI). Four GCTCVs and three important Philippine local varieties were assayed over two cropping seasons in a heavily *Foc*-infested field in the southern Philippines. While the commercially-grown varieties “Grand Naine” and “Lakatan” showed disease incidence up to 92%, the GCTCV varieties were largely resistant, with a disease incidence of 0–8%. Moreover, “Saba” (ABB) was completely resistant in the two seasons (Molina et al., 2016a). The TR4-susceptibility of African banana germplasm, generally known to be resistant to *Foc* R1, has been started to be evaluated. A collection of 14 genetically diverse EAHB and plantain varieties were evaluated in fields of China and the Philippines and found all resistant with disease incidence as low as 0–5%, except EAHB “Ibwi” which showed a disease incidence of 32% (Molina et al., 2016b).

When resistant varieties are not available, the management of FWB relies on an integrated approach. In the Canary Islands, for example, where FWB is caused by *Foc* STR4 and has a low prevalence, farmers try to contrast the pathogen by a number of palliative practices, sometimes based on empirical knowledge or deduced from basic research (Rodríguez Serrano, 2012; López-Cepero et al., 2014).

Soil amendment with calcium hydroxide or agricultural lime is practiced at the base of both diseased plants and symptomless neighboring ones with the aim to increase the pH and thus hinder *Foc* proliferation. The pH increase, however, may have deleterious effects on the physicochemical properties of soil, but probably the technicians and farmers choose the lesser evil. It has been observed that soil applications of CaCO_3_, Ca(OH)_2_, CaSO_4_, or Fe-EDDHA reduced *Foc* conidia germination and FWB severity, though the calcium amount was insufficient to change the soil pH. Nevertheless, this observation was not considered conclusive enough by the authors (Peng et al., 1999). A higher FWB incidence has been associated with low soil pH (Alvarez et al., 1981), but such observation has not been reproduced experimentally (da Silva Junior et al., 2000). Also, ammonia fumigation and biofertilization have been reported to reduce FWB incidence in a pot-experiment, with concurrent increases in soil pH, nutrient contents, and beneficial microbial community (Shen et al., 2019). Although no clear experimental evidence exists about the effectiveness against FWB of raising soil pH, it is known that *Foc* prefers low pH. For instance, a semi-selective agar medium widely used for isolation and enumeration of *Foc* from soil must be adjusted to pH 3.8–4.0 to favor *Foc* and hamper the growth of other fungi (Komada, 1975).

Adequate irrigation and fertilization regimes are also important to combat FWB. Waterlogging and acidification of nutritive solutions are generally avoided, and monthly treatments with zinc sulfate recommended. Stimulated by practical evidence that indoleacetic acid sprays combined with zinc supply reduced FWB, Hecht-Buchholz et al. (1998) observed ultrastructural changes of chloroplasts and mitochondria in banana leaves associated with the zinc-deficiency and, thus, with the FWB reduction. In a later work, the same research team confirmed the involvement of zinc nutrition in FWB development. Plants fertilized with a Zn-deficient solution were all diseased, whereas only 25% of plants treated with a normal zinc solution showed FWB symptoms (Fernández-Falcón et al., 2004). Overall, zinc seems to have a detrimental effect on several species of *Fusarium*, as observed in *F. moniliforme* (reduced fusarin C biosynthesis), in *F. oxysporum* f. sp. *radicis-lycopersici* (suppressed fusaric acid production) and *F. verticillioides* (reduced fumonisin production) (Jackson et al., 1989; Duffy and Défago, 1997; Savi et al., 2013). Similarly, silicon can decrease the intensity of several crop diseases (Datnoff and Rodrigues, 2015), including FWB (Fortunato et al., 2012b). This element may accumulate in the plant cell walls and act as a mechanical barrier, while it can also induce the phenylpropanoid pathway (e.g., lignin) and activate defense-related enzymes (e.g., chitinase, β-1,3-glucanase, phenylalanine ammonia-lyase, glucanase, peroxidase, polyphenoloxidase, etc.) (Smith et al., 2005; Fortunato et al., 2012a, 2014). Besides zinc and silicon, several other micro-nutrients like boron, iron, copper, and sodium, are known to affect FWB development (Qi et al., 2008; Sanjeev and Eswaran, 2008; Ji et al., 2012).

In the Canary Islands, it is also recommended (i) to increase the soil organic matter content above 3%, (ii) to avoid mulching for favoring soil aeration, (iii) to avoid high plant densities, and (iv) to apply plant resistance elicitors among other measures. Such practices arise from technical reports and, sometimes, from scientific articles (Alvarez et al., 1981; Aguilar et al., 2000; da Silva Junior et al., 2000; Rodríguez Serrano, 2012; López-Cepero et al., 2014). To the best of our knowledge, no detailed, long-term scientific studies have been conducted on the actual outcome of most of these practices, although they seem to help farmers to control FWB in a reasonable way.

Recently, Azkolain Olaondo (2016) investigated the use of biofumigation and soil solarization to manage FWB on a commercial farm in La Gomera island. However, results showed no differences in FWB incidence and severity among the treatments used (sheep manure or packing debris vs. control), although slight growth promotion was observed compared to the control. Moreover, solarization did not improve biofumigation treatments. An early trial from South Africa reported soil solarization as ineffective against FWB (Herbert and Marx, 1990). Nevertheless, in Indonesia, soil mulching with transparent polyethylene plastic for 10 months provided a 60% disease control until 14 months after planting. This treatment was superior to crop rotation with maize and bare soil treatment in terms of disease control and *Foc* TR4 suppression in the soil (Hermanto et al., 2012).

Crop rotation has provided attractive results in some cases. Rotation with rice reduced FWB to 8.1–17.6% after 1 year and to 0.8–6.3% after 2.5–3 years, compared to an initial level of 30–50%. *Foc* R4 population in the top layer of soil (20 cm) was undetectable after the treatment (Hwang, 1985). Furthermore, rotation with Chinese leek (*Allium tuberosum*) reduced FWB incidence and severity by 88–97% and 91–96%, respectively, and increased yield by 36–86% (Huang et al., 2012). *In vitro* assays showed that crude extracts of Chinese leek inhibit *Foc* R4 growth, suppress conidia proliferation and germination, and inhibit the activity of two cell wall degrading enzymes produced by *Foc*, polygalacturonase, and cellulase (Huang et al., 2012; Yang J. et al., 2015). Aqueous leachates and volatiles from Chinese leek exhibited strong inhibitory activity against *Foc*. Five volatiles including 2-methyl-2-pentenal and four organosulfur compounds (dimethyl trisulfide, dimethyl disulfide, dipropyl disulfide, and dipropyl trisulfide) were identified from the leaves and roots and found particularly active against the pathogen *in vitro* (Zhang et al., 2013). The pineapple-banana rotation has also given promising results. A 2-year crop rotation culminated with an 81% FWB reduction, and such control level was also attributed to changes in the bacterial and, more importantly, fungal communities in the soil (Wang B. et al., 2015). Lower disease control levels have been obtained by using rotations with maize, sugarcane, sunflower or eggplant (Hwang, 1985; Hermanto et al., 2012; Wang B. et al., 2015; Hong et al., 2017).

Biological (or reductive) soil disinfestation (BSD) has surfaced as another encouraging control means against FWB. Field experiments have shown that FWB can be reduced up to 82% in flooded soil incorporated with 0.5% rice straw (Huang et al., 2015c). The BSD simultaneously reduced the *Foc* inoculum in the soil and ameliorated the beneficial microbial communities, making the soil more suppressive to FWB (Huang et al., 2015a). Also, some microbes (e.g., *Clostridium* spp.) producing organic acids toxic for *Foc* (acetic, butyric, isovaleric and propionic acids) were found more abundant after BSD (Huang et al., 2015b).

## Biological Control Agents and Their Modes of Action: Actual and Effective Tools to Confront Fusarium Wilt of Banana

From a historical perspective, biocontrol of FWB has been studied for more than 70 years (Thaysen and Butlin, 1945). Studies have reported that disease suppressive sites showed microbial communities displaying higher richness and diversity (Shen et al., 2015b; Köberl et al., 2017), and possibly a higher number of antagonistic members, as observed for streptomycetes (Kinkel et al., 2012; Jauri et al., 2017). Moreover, differences in the composition of these communities correlated with the disease suppressiveness (abundance of *Acidobacteria*) or conduciveness (abundance of *Bacteroidetes*) of soil (Shen et al., 2015b). The manipulation of banana rhizosphere microbiota by the introduction of well-characterized antagonists alone or in combination with organic amendments (bio-organic fertilizers) has already yielded very promising results against *Foc* TR4 in China (Shen Z. et al., 2013; Shen et al., 2015a; Xue et al., 2015). This strategy also leads to changes in the structure and composition of the microbial community that can be harnessed for more effective control of FWB (Shen et al., 2015a; Fu et al., 2016b).

**Figure 4:**
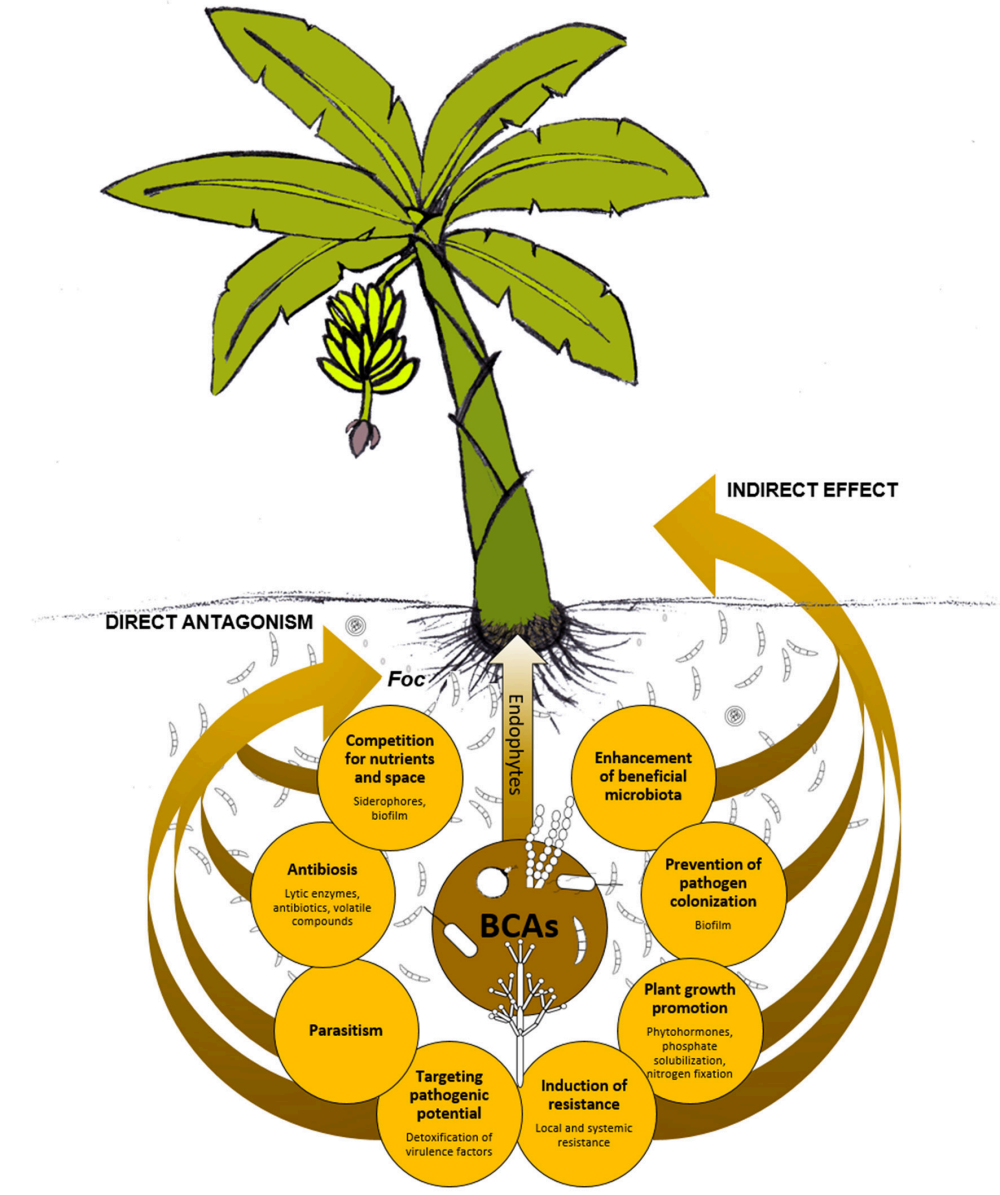
Possible modes of action of biological control agents (BCAs). Beneficial microorganisms can exhibit direct antagonism against *Fusarium oxysporum* f. sp. *cubense* (*Foc*) and can affect the plant physiology and/or the microbiota with a consequent, indirect effect against the pathogen.

**Figure 5:**
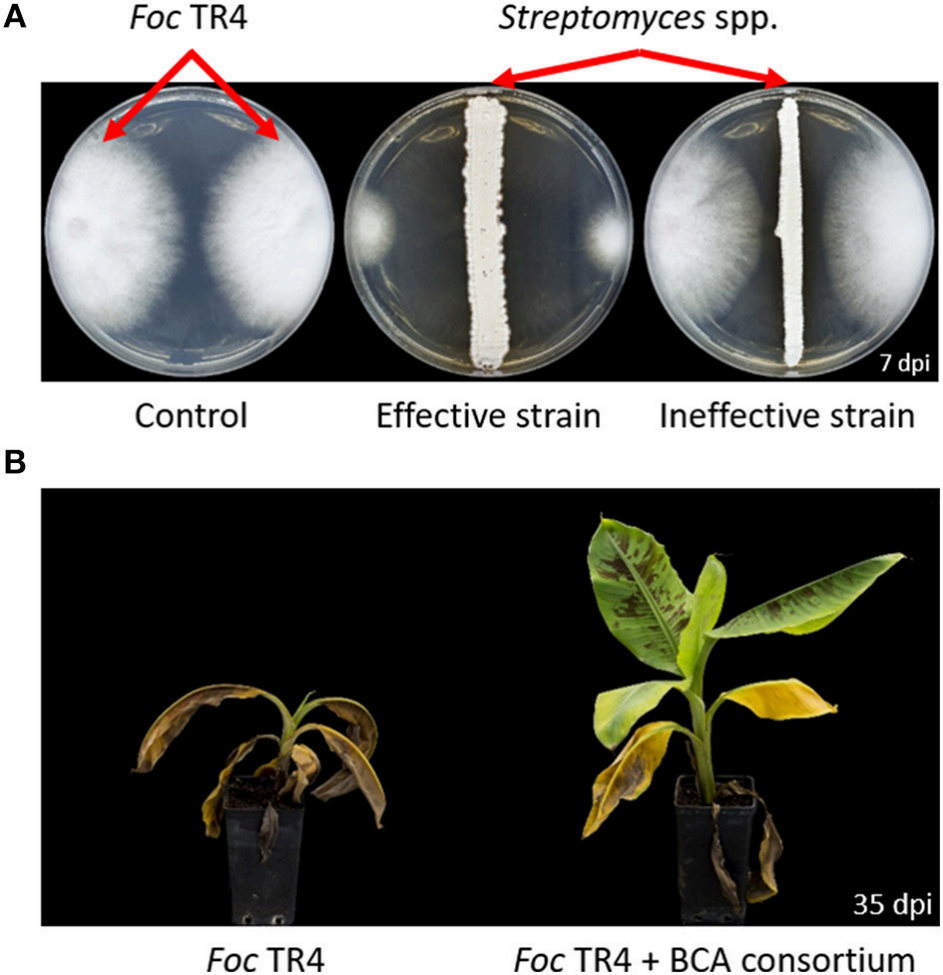
Effectiveness of biological control agents against *Fusarium oxysporum* f. sp. *cubense* (*Foc*). The dual culture method **(A)** allows testing the *in vitro* antifungal activity of metabolites produced by biological control agents (BCAs). Pot experiments **(B)** provide first evidence for the *in planta* efficacy of BCAs.

The mechanisms underlying the BCAs' biocontrol activity are many and variegated (Narayanasamy, 2013; Singh, 2014). It is essential to know the BCAs' modes of action, including weakness and requirements, in order to exploit their potential for disease management in the most effective manner. Also, the combination of BCAs with different modes of action might result in a better biocontrol due to additive, or even synergistic, interactions between BCAs (Parnell et al., 2016; De Vrieze et al., 2018). Figure 4 schematizes how BCAs can act directly or indirectly against *Foc*. Direct antagonism may be due to antibiosis (e.g., antibiotics, lytic enzymes, volatile organic compounds, etc.), parasitism, or competition (for space and/or nutrients). Induction of plant local/systemic resistance, plant growth promotion, or changes of soil/plant microbiota in favor of more beneficial microbial taxa are typical mechanisms that indirectly act against the pathogen, or at least contribute to reducing the infections or the disease. Antibiosis is one of the primary mechanisms possessed by BCAs. Indeed, initial *in vitro* selection of new BCAs often relies on the evaluation of the sole anti-microbial activity against the pathogen (Figure 5A), while other mechanisms are studied later on, possibly once the BCA effectiveness is demonstrated at least under controlled conditions (Figure 5B).

### Literature Overview Shows That Biocontrol of Fusarium Wilt of Banana Is a Widely Studied Research Topic

In order to understand the relevance of the research on biological control of FWB, we searched the keywords “*Fusarium cubense*” or “Panama disease” in the title and abstract of articles indexed by the CAB Direct database (CAB Direct, 2018). The search yielded a comprehensive overview of the literature from 1970 to date. Amongst 735 retrieved articles, 367 were focused on disease management and, with 182 articles (ca. 25% of the total), biological control represents the largest research sub-topic on FWB (Figure 3). Records of FWB presence, distribution or impact on the crop productivity rank second for their abundance (119 articles), suggesting how much the disease is feared worldwide (Figure 3). With 123 retrieved articles, breeding for resistance is the third relevant FWB-related research topic (17% of the total articles), which in our article categorization included conventional breeding, screening for resistance, development, and evaluation of mutant and transgenic bananas. Therefore, biological control and host genetic resistance have been considered the most important strategies for the management of FWB. It should be noted that conventional breeding in banana is particularly laborious because cultivated varieties are polyploid; hence, diploid parents must be used (or generated) for crosses, and then promising progeny must be polyploidized. In the late 70's, the research on breeding was much more intense than that on biological control, which increased only since 2000s (Supplemental Figure 1). At least 143 articles deal with BCAs. Endophytes and strains of *Trichoderma* spp., *Pseudomonas* spp., and *Bacillus* spp. have been the most studied BCAs, followed by arbuscular mycorrhizal fungi. *Streptomyces* spp. have been investigated mainly *in vitro*, with very few *in planta* experiments, and none conducted in the field (Figures 3 and Supplemental Figure 2). In 39 papers, the effects of natural products against *Foc* is showed and, interestingly, 12 articles published since 2011 report studies on the efficacy of *A. tuberosum* extracts (Supplemental Figure 1). The remaining articles are focused on the interactions of *Foc* with other microorganisms and its population biology, where the “-omics” approach has been used since the 2010s. Experiments conducted under field, pot and *in vitro* conditions are listed in Tables 1, 2 and Supplemental Table 1, respectively, and their by-taxon abundance is summarized in Supplemental Figure 2.

**Table 1:** List of biocontrol field trials against Fusarium wilt of banana.

**Biocontrol agents**	**Mode of application**	***Foc* race**	***Foc* inoculum**	**Best disease control obtained (%)**	**Highest yield increase obtained (%)**	**Relevant remarks**	**References**
***Endophytes***							
*Pseudomonas aeruginosa* FJAT-346-PA				82		Colonization study with antibiotic-marked strains	Yu C. et al., 2010
*Acremonium* sp. Q34	Root dripping into the fermentation broth	4	10^5^ conidia mL^−1^	71		Strain isolated from disease-free *Kandelia candel*	Liu and Lu, 2013
*Burkholderia cenocepacia 869T2*	Root dipping (OD_600_ = 0.6–0.7)	TR4	Natural infestation	86	11	Growth-promoting effects	Ho et al., 2015
*Pseudomonas* *putida* C4r4, *Achromobactrum* sp. Gcr1, *Rhizobium* sp. Lpr2, *Bacillus flexus* Tvpr1	Talc powder formulation of bacterial consortia (10^8^ cells g^−1^)	1	Natural infestation	42.2	214	Combined effect with rhizospheric isolates (*B. cereus* Jrb1, *P. putida* Jrb2, *Bacillus* sp. Jrb6 and Jrb7)	Thangavelu and Gopi, 2015b
*Serratia marcescens* ITBB B5-1	Pre-planting soil drenching	4	Pre-planting soil drenching (10^6^ conidia mL^−1^)	70		Isolated from the rubber tree	Tan et al., 2015
*Pseudomonas fluorescens* Pf1, *Bacillus subtilis* EPB 10 and EPB 56	*In vitro* co-culturing of plants with bacteria		*In vitro* co-culturing of plants with *Foc* after bacteria inoculation			Increased leaf nutrient status and enhanced growth, bunch yield and fruit quality	Kavino et al., 2016
*Pseudomonas fluorescens* Pf1, *Bacillus subtilis* EPB 10 and EPB 56	Root dipping (3·10^10^CFU mL^−1^)	1	Injection into the corm (10^6^ conidia mL^−1^)	78	119		Kavino and Manoranjitham, 2018
*Fusarium oxysporum* CAV553 and CAV255, *Pseudomonas fluorescens* WCS417	Root dipping and soil drenching (10^6^ conidia mL^−1^)	STR4	Natural infestation	0		Endophytes from healthy micropropagated Cavendish banana roots (South Africa)	Belgrove et al., 2011
*Trichoderma asperellum* Prr2 (endophyte), *Trichoderma* sp. NRCB3 (rhizospheric)	Colonized rice chaffy grains	1	Natural infestation	47	45	Growth-promoting effects	Thangavelu and Gopi, 2015a
***Trichoderma*** **spp**.							
*T. viride*	Root dipping (10^6^ conidia mL^−1^), followed by application of colonized wheat bran:saw dust mixture		Natural infestation	75	60		Raguchander et al., 1997
*T. viride* NRCB1	Colonized rice chaffy grains (1·10^31^ CFU g^−1^) + 5% jaggery solution		Colonized sand:maize mixture	80		Induction of peroxidase, phenylalanine ammonia lyase, and total phenolic content	Thangavelu and Mustaffa, 2010
*T. harzianum* TH UH and TH13		TR4		0			Wibowo et al., 2013
*T. harzianum* (ECO-T®)	Soil inoculation in the nursery			68		*Foc* reduction: 68% in Humic Nitisol, 6% in Rhodic Ferralsol	Mukhongo et al., 2015
***Pseudomonas*** **spp**.							
*P. fluorescens*	Root dipping (10^6^ conidia mL^−1^), followed by wheat bran application		Natural infestation	77	68		Raguchander et al., 1997
*P. fluorescens* Pf1	Different combinations of paring and pralinage with a *P. fluorescens* formulation (2.5·10^8^ CFU g^−1^), soil application, and capsule application		Natural infestation	80.6		Two field trials. Pairing and pralinage with *P. fluorescens* formulation + capsule application at 3 and 5 months after planting gave the best results	Raguchander et al., 2000
*P. fluorescens* Pf1	Soil inoculation at transplanting and post-planting with a talc-based powder formulation			100	68		Rajappan et al., 2002
*P. fluorescens* Pf1	Soil application combined with *Bacillus subtili*s TRC 54 and a plant extract-based fungicide		Natural infestation	75		Also, 64% FWB reduction in greenhouse	Akila et al., 2011
*P. fluorescens* Pf1	4 L ha^−1^ of liquid formulation (9·10^8^ CFU mL^−1^)			60	47	Three field trials. Also, 41.3–89% reduction of *Helicotylenchus multicinctus*	Selvaraj et al., 2014
***Bacillus*** **spp**.							
*B. subtilis* TR21	Root dipping			73.9			Yu G. et al., 2010
*B. subtili*s TRC 54	Soil application combined with *Pseudomonas fluorescens* Pf1 and a plant extract-based fungicide		Natural infestation	75		Also, 64% FWB reduction in greenhouse	Akila et al., 2011
*Bacillus* spp. (PHC Biopak®)	Soil inoculation in the nursery			50		*Foc* reduction: 50% in Vertisol, 47% in Humic Nitisol	Mukhongo et al., 2015
*B. amyloliquefaciens* NJN-6	Colonized bio-organic fertilizer			68.5	100	Isolated from suppressive soil	Xue et al., 2015
*B. amyloliquefaciens* W19	Colonized bio-organic fertilizer (10^9^ CFU g^−1^)		Natural infestation	44.4	34.5	Banana root exudates Enhanced colonization	Wang et al., 2016
**Non-pathogenic** ***Fusarium oxysporum***							
*F. oxysporum* Ra-1, Ro-3	Colonized sand:maize mixture	1	Colonized sand:maize mixture	84			Thangavelu and Jayanthi, 2009
*F. oxysporum* UPM31P1	Colonized peat:perlite:oats (2:1:2 vol.) (10^6^ UFC g^−1^)	TR4		0			Ting et al., 2009c
**Arbuscular mycorrhizal fungi**							
*Glomus mosseae, Trichoderma harzianum*	Inoculation at transplanting	1	Colonized sorghum grains (1.5·10^6^ CFU g^−1^)	68 (measured by ELISA)	75	Growth promotion	Mohandas et al., 2010
*Glomus clarum*	Soil inoculation in the nursery			23		Ineffective when *G. clarum* was combined with *Pseudomonas putida* and *Trichoderma asperellum*	Lin et al., 2012
Rhizatech®	Soil inoculation in the nursery			55		*Foc* reduction in Humic Nitisol on cv. Gros Michel	Mukhongo et al., 2015
**Other microorganisms**							
*Serratia marcescens*	Bentonite and kaolin formulations	TR4				Bentonite performed better. In bentonite formulation, PABA should be omitted, while NFSM, and sucrose levels should be optimized	Ting A.S.Y. et al., 2011

**Table 2:** List of biocontrol pot-experiments against Fusarium wilt of banana.

**Biocontrol agents**	**Mode of application**	***Foc* race**	***Foc* inoculum**	**Best disease or *Foc* UFC reduction (%)**	**Relevant remarks**	**References**
***Endophytes***						
*Streptomyces griseorubiginosus* S96	Root dipping (10^6^ spores mL^−1^)	4	10^4^ conidia mL^−1^	47	Siderophore-producing strain, selected from 131 banana roots-endophytic actinomycetes	Cao et al., 2005
*F. oxysporum* BRIP 29089, 29093 and 45952	Colonized ground millet	1, STR4	Colonized ground millet	75 (R1) 67 (R4) (vascular discoloration)	Obtained from banana roots in suppressive soil	Forsyth et al., 2006
*Burkholderia* spp. AB202 and AB213, *Herbaspirillum* spp. BA227 and BA234	Root dipping (5·10^7^ CFU mL^−1^)			97 (CFU)	Isolated from roots and stem of pineapple and banana	Weber et al., 2007
Endophytic bacteria (mainly γ-Proteobacteria)	Crude endophytes inoculum (7.1 log CFU g^−1^)	4	10^5^ conidia mL^−1^	67	Growth-promoting effects	Lian et al., 2009
*Erwinia chrysanthemi* E353		4		60.67	Endophytic strain from a healthy banana plant in a *Foc*-infested field	Yin et al., 2009
*Penicillium citrinum*	Soil drenching (10^6^ conidia mL^−1^)	4	10^6^ UFC mL^−1^	2	Host defense response	Ting et al., 2012
*Pseudomonas fluorescens* Pf1, *Bacillus subtilis* EPB 10, and EPB 56	*In vitro* co-culturing of plants with bacteria		*In vitro* co-culturing of plants with *Foc* after bacteria inoculation			Kavino et al., 2014
*Trichoderma* sp. TJ5	Root dipping (10^6^ CFU mL^−1^)	1	Soil inoculation (10^6^ CFU mL^−1^)	62.5	Plant growth promotion	Caballero Hernández et al., 2013
*T. asperellum*		1				Chaves et al., 2016
***Trichoderma*** **spp**.						
*T. viride*	Soil inoculation					Shamarao et al., 2001
*T. harzianum*	Soil inoculation	4		0	*T. harzianum* + Ca(NO_3_)_2_	Ting et al., 2003
*T. viride*	Colonized organic amendments				Neem cake, groundnut cake, *Pongamia* cake	Satheesh and Venu, 2004
*T. viride*	Colonized corn grits		Colonized corn-meal:sand	81.76	Abaca (*Musa textilis*)	Bastasa and Baliad, 2005
*T. harzianum* A34	Soil inoculation (8·10^9^ UFC g^−1^)	4	Naturally infested soil	95	Plantains	Pérez Vicente et al., 2009
*Trichoderma* sp. TR76	Soil drenching (10^6^ UFC ml^−1^)			41		Hima and Beena, 2016
*Trichoderma* spp. T22 and T5	Soil drench (10^7^ spores mL^−1^)	4	Colonized millet seeds	62	Isolated from suppressive soil	Nel et al., 2006
*T. viridae*	Root dipping and soil drenching					Pushpavathi et al., 2017
*T. asperellum* PZ6	Root-injury irrigating method	4	Root-injury irrigating method	48	Plant growth promotion	Qin et al., 2017
***Pseudomonas*** **spp**.						
*P. fluorescens* Pfcp	Root dipping (10^8^ CFU mL^−1^)	1, 4	10^8^ conidia mL^−1^	80	Less severe wilting and internal discoloration. Improved root growth and enhanced plant height in *M. balbisiana*	Sivamani and Gnanamanickam, 1988
*P. fluorescens*	Root dipping (10^8^ UFC mL^−1^)					Shamarao et al., 2001
*P. fluorescens* Pf10	Soil inoculation			50	Detoxification of fusaric acid	Thangavelu et al., 2001
*P. fluorescens* Pf10	Soil drenching (10^9^ CFU mL^−1^)	1	Colonized sand:maize mixture		Induction of defense enzyme and phenolics	Thangavelu et al., 2003
*P. fluorescens* Pfm	Talc powder formulation (10^8^ CFU g^−1^)	1	10^6^ conidia mL^−1^	50 (vascular discoloration)	Enzymatic activity assay	Saravanan et al., 2004a
*P. fluorescens* Pfm. Pf1, Pf2, and Pf3	Talc powder formulation (10^8^ CFU g^−1^)	1		7.4 (spore germination)	Rifampicin resistant strain of *P. fluorescens*	Saravanan et al., 2004b
*P. fluorescens*	Colonized charcoal (10^8^ CFU mL^−1^)		Colonized sorghum seeds (9.2·10^4^ CFU mL^−1^)	72	Immunolocalization of both organisms in banana roots	Mohandas et al., 2004
*P. aeruginosa* FP10	Inoculation of *in vitro* plants				Plant growth promotion	Ayyadurai et al., 2006
*P. fluorescens*	Root dipping and soil drenching					Pushpavathi et al., 2017
***Bacillus*** **spp**.						
*B. subtilis*						du Plessis, 1994
*B. thuringiensis*	Root dipping (10^6^ CFU mL^−1^)					Shamarao et al., 2001
*B. licheniformis* C-4	Root dipping					Sun and Wang, 2009
*B. subtilis* KY-21	Soil drenching (5·10^5^ CFU mL^−1^)	4	Soil drenching (5·10^5^ CFU mL^−1^)	33	Induction of defense-related enzymes	Sun et al., 2011
*B. subtilis* N11	Colonized bio-organic fertilizer			82	Biofilms formation and enhancement of root elongation and differentiation zones	Zhang et al., 2011
*Bacillus* spp. RZ-1, 3, 10, 34, 35, 60, 69, and 76	Root dipping (OD_540_ = 0.5)		Soil drenching (10^5^ CFU mL^−1^)	16	Also, a dual effect on mortality and motility of *Meloidogyne javanica* second stage juvenile	Ribeiro et al., 2012
*B. amyloliquefaciens* W19	Colonized bio-organic fertilizer (10^9^ CFU g^−1^)		Naturally infested field soil (1.5·10^4^ CFU g^−1^)	77	Antifungal lipopeptides	Wang et al., 2013
*B. amyloliquefaciens* WJ22	Colonized bio-organic fertilizer (3·10^8^ CFU g^−1^)		Naturally infested field soil (1·10^3^ CFU g^−1^)	75.7	Antifungal lipopeptides	Wang J. et al., 2015
**Non-pathogenic** ***Fusarium oxysporum***						
*F. oxysporum* CAV 255 and CAV 241	Soil drench (10^7^ spores mL^−1^)	TR4	Colonized millet seeds	87.4	Obtained from suppressive soil	Nel et al., 2006
***Streptomyces*** **spp**.						
*Streptomyces* sp. g10	Soil drenching (10^8^ CFU mL^−1^)	4	10^4^ or 10^6^ conidia mL^−1^	47	Effective against *Foc* at 10^4^ conidia mL^−1^ but not at 10^6^ conidia mL^−1^	Getha et al., 2005
*S. lunalinharesii* B-03	Fermentation broth	4	10^6^ CFU mL^−1^	73	Effective *in vitro* against nine pathogens	Zhou et al., 2017
8 actinomycetes	Fermentation broth	4	1.85·10^6^ conidia mL^−1^	87	Selected from 139 isolates. Effective *in vitro* against several *F. oxysporum* ff. spp.	Qin et al., 2010
**Arbuscular mycorrhizal fungi**						
*Glomus intraradices, Glomus* spp.	Soil inoculation in the nursery				Plant growth promotion	Jaizme-Vega et al., 1998
*Glomus fasciculatum*	Soil culture (500 chlamydospores)			43 (CFU)	Increased cell size and number. More total insoluble polysaccharides, total proteins, and total nucleic acids	Habeeba et al., 2003
*Gigaspora margarita*	Soil inoculation in the nursery				FWB reduction dependent on AMF and *Foc* inoculum concentrations	Borges et al., 2007
Native arbuscular mycorrhizal fungi	3.5·10^3^ or 7·10^3^ kg^−1^		10^6^ conidia mL^−1^		More mycorrhiza in plants treated with a biofertilizer rather than three concentrations of Hoagland solution	Sampaio et al., 2012
**Other microorganisms**						
Rhizospheric strains FB5, FB2, T2WF, T2WC, and W10	Root dipping	4		81		Yang et al., 2006
Bacteria 0202 and 1112		4				Wang et al., 2011
*Paenibacillus* spp. RZ-17, and RZ-24	Root dipping (OD_540_ = 0.5)		Soil drenching (10^5^ CFU mL^−1^)	16	Dual effect on mortality and motility of *Meloidogyne javanica* second stage juveniles	Ribeiro et al., 2012
Marine rhizobacteria YS4B1, YS1A3, YS2A5					Isolated from mangrove rhizosphere. Effective also against *Ralstonia solanacearum* and *Mycosphaerella fijiensis*	Bonsubre et al., 2016
*F. oxysporum* f. sp. *cubense* (dead)			10^4^ CFU mL^−1^	100		Chand et al., 2016

**Figure 6:**
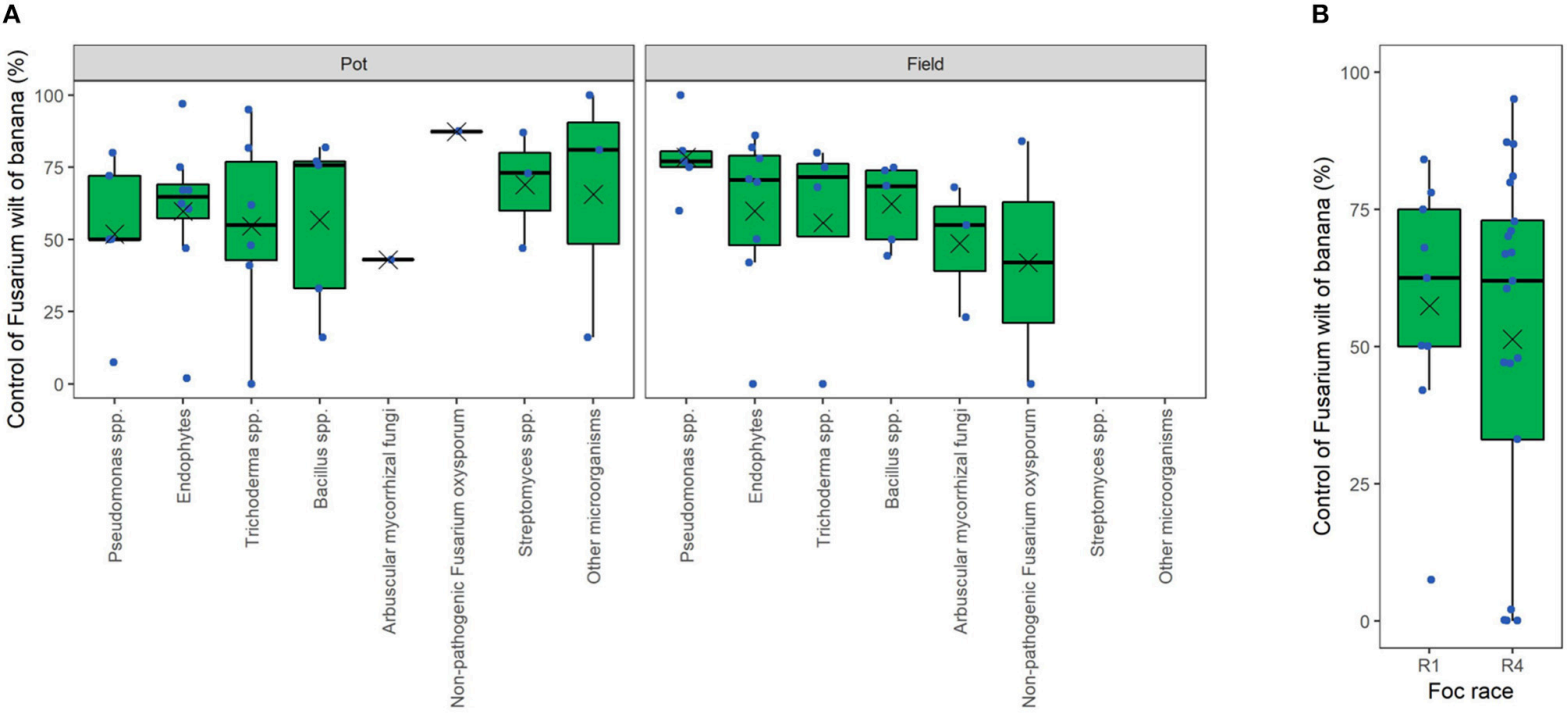
Overview of the efficacy of biological control agents against Fusarium wilt of banana. Data of pot and field trials separated by microbial groups **(A)** or by *Fusarium oxysporum* f. sp. *cubense* (*Foc*) races **(B)**. Dots correspond to the best control levels (*viz*. reduction of disease incidence or severity) obtained in each scientific article in the literature. In the boxes, the mean is reported as a cross and the median as a horizontal line.

Data mining from the literature allowed us to infer on the potential and actual efficacy of several microbial genera in the control of FWB. In particular, from each retrieved article we mined the best FWB control value obtained, but not all the data. Although the experiments are not so numerous, plotting these data provides some evidence for microbial genera more prone to control FWB than others (Figure 6A). Interestingly, we realized that several biocontrol trials reached the field stage, and results even showed high effectiveness. In the field, FWB was controlled up to 77% (median of 5 articles) by *Pseudomonas* spp. strains, and up to 71% by several endophytic strains (8 articles) and other *Trichoderma* spp. strains (4 articles) (Figure 6A). Lower biocontrol was obtained with *Bacillus* spp. (69% as the median of 5 articles), arbuscular mycorrhizal fungi (55%, 3 articles), and non-pathogenic *Fusarium* strains (42%, 2 articles), whereas most studies on *Streptomyces* spp. have been limited to *in vitro* conditions. Biocontrol under field conditions has been often reported to be less effective than under (semi)controlled, pot experimental conditions. Surprisingly, a different scenario appears for FWB biocontrol. In fact, pot- (median = 65) and field-experiments (median = 70) resulted substantially similar in terms of disease control efficacy. Moreover, articles dealing with *Pseudomonas* spp. reported on average a higher efficacy in the field (median = 77%) than in pot-experiments (median = 50%), as also observed for endophytes, *viz*. 71% in the field and 65% in pots. Non-pathogenic *Fusarium* strains, however, resulted more effective in pot-experiments (median = 87%) than in the field (median = 42%). Finally, *in planta* effectiveness of BCAs was not dependent on the target *Foc* race (R1 and R4), meaning that *Foc* R1 and R4 showed overall comparable sensitivity to BCAs (median disease control efficacy of 62%; Figure 6B).

### Endophytes: The Help From Inside

All plants harbor a huge diversity of beneficial or neutral microorganisms living inside their tissues without causing any deleterious effect in the host (reviewed by Hardoim et al., 2015). Since beneficial endophytes can promote plant fitness and growth through a range of different mechanisms (i.e., phytohormones synthesis, nitrogen fixation, phosphate solubilization, induction of defense responses, alleviation of abiotic stress by reducing ethylene level, etc.) (Compant et al., 2016), they have a considerable agro-biotechnological potential yet to be fully exploited (Mercado-Blanco and Lugtenberg, 2014, and references therein). Endophytes have found evolutionary solutions to live within the plant interior (i.e., nutrient availability, evading/modulating host defense responses, etc.), where they can also deploy biocontrol activity against pathogens. Thus, an increasing number of studies are available on the isolation, characterization, and assessment as BCAs of specific culturable members of indigenous endophytic communities. Endophytic bacteria and fungi from banana plants have received early attention as BCAs against *Foc* and other biotic constraints (Ortiz and Pocasangre, 2012; Niere et al., 2014). Although many of them belong to genera that will be described in more detail in the following sections, we would like to summarize some reports focused on endophytes to highlight the importance of this special group of the plant-associated microbes.

It is interesting that many experiments with endophytes against FWB have been conducted in the field during the last years. One of the early research showed how *P. aeruginosa* FJAT-346-PA reduced FWB by 82–84% in semi-field and field conditions. The strain was proved to colonize the roots and stems of banana and to promote the plant growth (Yu C. et al., 2010). Cao et al. (2004) reported on the actinomycete communities found in the interior of leaves and roots of both healthy and diseased banana plants. Most of the isolates were *Streptomyces griseorubiginosus*-like strains. Isolates displaying antagonistic activity against *Foc* originated from roots of healthy plants, whereas no difference in this phenotype was reported between antagonists isolated from healthy and wilted leaves. *In vitro*, the antagonism of *S. griseorubiginosus* S96 was lost when FeCl_3_ was introduced in the Petri plates, suggesting that it relies on siderophore production. *In vivo*, FWB severity (R4) was reduced by 47%, and plant fresh weight increased in plantlets treated with strain S96 compared to the control (Cao et al., 2005). Similarly, three endophytic *Bacillus* spp. isolated from different *Musa* cultivars in Brazil showed antagonistic activity against *Foc* and *Colletotrichum guaranicola* (Souza et al., 2014). More recently, endophytic bacteria from the classes Actinobacteria (genera *Arthrobacter, Brevibacterium, Corynebacterium, Curtobacterium, Kocuria, Kytococcus, Micrococcus, Naumanella, Rothia*, and *Tessaracoccus*), α- and γ-Proteobacteria (*Brevundimonas, Enterobacter, Klebsiella, Pseudomonas, Serratia*, and *Sphingomonas*) and Firmicutes (*Bacillus* and *Staphylococcus*) were isolated from the shoot-tips of banana cv. Grand Naine (Sekhar and Pious, 2015). From the collection generated in this study, strains of *Pseudomonas aeruginosa, Klebsiella variicola*, and *Enterobacter cloacae* showed variable antagonistic activity against *Foc*, but their potential as effective BCAs remained to be proved. Other studies reported on fungal and bacterial endophytes originating from plants other than banana such as weeds and medicinal plants (Ting et al., 2009b) or *Capsicum frutescens* (He H. et al., 2002), making them interesting reservoirs of *Foc* antagonists worth to be explored. Finally, some studies go a step forward, exploring the potential mechanism involved in the antagonistic effect. For instance, Ting et al. (2010) investigated the role of volatiles produced by several fungal endophytes in the growth inhibition of *Foc* R4. Even if with inconsistent disease control, the same authors also suggested the induction of host resistance as a mechanism involved in FWB control mediated by *Penicillium citrinum* BTF08 isolated from banana internal stem tissues (Ting et al., 2012).

Interestingly, some of the reports were focused on the inoculation of BCAs at the *in vitro* propagation stage of banana plants. Banana tissue culturing is widely used as a source of pathogen-free planting material. Since banana plantlets produced by these micropropagation schemes are more susceptible to diseases due to the absence of the natural-associated microbiota, the reintroduction of endophytic and rhizospheric microorganisms to protect the plant against subsequent challenges by *Foc* is an approach that yielded promising results (Kavino et al., 2014, 2016; Kavino and Manoranjitham, 2018). The *in vitro* co-culturing of banana plants with *Pseudomonas fluorescens* Pf1, *Bacillus subtilis* EPB 10 and EPB 56 provided a successful control of FWB in the field, combined with increased leaf nutrient status, vegetative growth, bunch yield and fruit quality (Kavino et al., 2016). In two other field trials, the endophyte and rhizobacteria strain led to a FWB control of 78% and a significant higher bunch weight (Kavino and Manoranjitham, 2018). Furthermore, this strategy was also proved to be successful when inoculating banana tissue culture plantlets with a mixture of naturally-occurring uncultivated endophytes from healthy banana plants of a commercial plantation (Lian et al., 2009). In this latter study, the re-introduction of endophytes to the banana tissue culture led to a significant reduction of wilt disease (67%) caused by *Foc* R4 (artificial inoculation) and growth promotion under greenhouse conditions. Ten non-pathogenic *F. oxysporum* isolates obtained from healthy micro-propagated “Cavendish” banana roots were able to significantly reduce FWB under greenhouse conditions, but none of them nor *P. fluorescens* WCS417 gave protection from the disease (STR4) in the field (Belgrove et al., 2011). Some researchers tried to have more chances to obtain effective BCAs by isolating from FWB-suppressive soil, e.g., non-pathogenic *F. oxysporum* strains (Forsyth et al., 2006), or even from healthy banana plants located in *Foc*-infested soil, e.g., *Erwinia chrysanthemi* E353 (Yin et al., 2009).

Furthermore, endophytes effective against FWB did not originate only from banana plants. For instance, Ho et al. (2015) isolated *Burkholderia cenocepacia* 869T2 from surface-sterilized vetiver grass (*Chrysopogon zizanioides*) roots. Banana tissue culture plantlets inoculated with 869T2 showed a lower disease incidence caused by *Foc* TR4 (86% incidence reduction) as well as significant plant growth promotion under field conditions. The endophytic strain *Serratia marcescens* ITBB B5-1 was isolated from the rubber tree (*Hevea brasiliensis*) (Tan et al., 2015). A sharp reduction in disease severity caused by *Foc* R4 was scored under both greenhouse (79%) and field conditions (70%) upon inoculation of banana plants with this strain. Moreover, chitinase and glucanase activities were suggested to be involved in its antifungal activity (Tan et al., 2015). Endophytic diazotrophic bacteria (strains of *Burkholderia* sp. and *Herbaspirillum* sp.) isolated from roots and stems of pineapple (Weber et al., 2007), and an *Acremonium* sp. strain isolated from *Kandelia candel* (Liu and Lu, 2013) are additional examples of promising biocontrol and biofertilizer candidates for the banana crop that originate from a different host. Finally, effective control of FWB and production increase (number of banana hands and bunch weight) was reported under field conditions when different combinations of endophytic (*P. putida* C4r4, *Achromobactrum* sp. Gcr1, *Rhizobium* sp. Lpr2, and *B. flexus* Tvpr1) and rhizospheric bacteria (*B. cereus* Jrb1, *P. putida* Jrb2, *Bacillus* sp. Jrb6, and Jrb7), in this case isolated from different banana accessions, were applied to a naturally infested soil (Thangavelu and Gopi, 2015b). In another field trial, combined applications of the endophytic *T. asperellum* prr2 together with the rhizospheric *Trichoderma* sp. NRCB3 resulted in a 47% reduction of FWB incidence and a 45% increase of the bunch weight (Thangavelu and Gopi, 2015a).

### *Bacillus* spp.: The Endospore-Forming Bacteria

The use and number of *Bacillus* spp. strains displaying suppressive effect against plant diseases caused by soil-borne phytopathogens has been increased rapidly, and a large body of knowledge on the biocontrol mechanisms involved as well as on their application and effectiveness under diverse conditions is available for the interested reader (Fira et al., 2018; Aloo et al., 2019). The spore-forming ability of *Bacillus* species confers them an important advantage over other beneficial microorganisms in the field of biological control. On the one hand, this capability enables these bacteria to endure adverse environmental conditions. On the other hand, and from the agro-biotechnological point of view, it favors the development and manufacturing of commercial formulations more stable over time. In addition, many *Bacillus* species show rapid growth rates and the ability to synthesize a large number of secondary metabolites which play a key role in the antibiosis against many deleterious microorganisms (Radhakrishnan et al., 2017; Fira et al., 2018). Some species, such as *B. subtilis*, are also able to produce volatile organic compounds (VOCs), which are important sometimes for plant growth promotion and the activation of plant defense mechanisms by triggering induced systemic resistance (Raaijmakers et al., 2010; Cawoy et al., 2014). *Bacillus*-mediated plant growth promotion can also be due to the capacity to promote phytohormone (i.e., gibberellic acid and indole-3-acetic acid) biosynthesis, thereby enhancing nutrient uptake ability in the host and stimulating plant defense responses against biotic and abiotic stresses (Chen et al., 2007; Harman, 2011). Besides the production of antibiotics and the elicitation of systemic resistance in plants against pathogens, *Bacillus* species are also able to produce lytic enzymes like chitinase and β-1,3-glucanase, involved in the degradation of the fungal cell wall (Kumar et al., 2012). Considering their versatility, the combination of different *Bacillus* spp. strains (or with other BCAs) displaying different biocontrol mechanisms appears as an interesting approach to improve biocontrol effectiveness under different cropping scenarios and environmental conditions.

*Bacillus* spp. are commonly found in the banana rhizosphere (Xue et al., 2015), and many members of this genus have already been investigated as BCAs of diverse *Fusarium*-induced plant diseases (Khan et al., 2017). Among them, representatives of *B. subtilis, B. amyloliquefaciens, B. pumilus* and *B. thuringiensis* are found in the literature. *Bacillus subtilis* is well known for its antagonistic activity against several fungal and bacterial plant diseases. Its biocontrol activity is mainly attributed to antibiotic production (Cawoy et al., 2015), and its enzymatic products are highly active against many fungal pathogens. The biocontrol effect of the plant endophytic *B. subtilis* strain TR21 against FWB was investigated in Brazilian fields, and promising results (74% effectiveness) were reported (Yu G. et al., 2010). Likewise, *B. subtilis* strain N11 isolated from the rhizosphere of a healthy banana plant showed biocontrol activity in pot experiments under greenhouse conditions (Zhang et al., 2011). Addition of 10% (v/v) of culture filtrate of the endophytic *B. subtilis* strain EBT1 to the plant growth medium increased bud multiplication, plantlet weight, pseudo-stem height, and conferred resistance to plantlets against *Foc* conidia and toxin (Yang et al., 2010). *Bacillus subtilis* strain B25, isolated from banana rhizosphere soil in Hainan, is another example of an effective antagonist, not only against *Foc*, but also against other plant pathogenic fungi including *Corynespora cassiicola, Alternaria solani, Botrytis cinerea*, and *Colletotrichum gloeosporioides* (Tan et al., 2013; Yu et al., 2016). Results on its capability to control FWB under greenhouse and field conditions can be found in the literature, although they are not easily accessible (Liu, 2011). The antifungal protein of B25, identified as a disease-resistance protein, produced mycelium and spore tumescence and abnormal growth of the pathogen (Tan et al., 2013). The chitinolytic and heat tolerant strain *B. subtilis* TSA3 showed *in vitro* inhibition of *Foc* growth although effectiveness *in planta* and under field conditions has not been demonstrated yet (Nawangsih and Purba, 2013). Similarly to previous examples, *B. subtilis* strain S-1 not only inhibited *in vitro Foc* growth but also antagonized fungal pathogens such as the *formae speciales lycopersici, vasinfectum* and *niveum* of *F. oxysporum*, as well as *Curvularia lunata, C. gloeosporioides, Verticillium dahliae*, and *Gibberella zeae* (Sun et al., 2008).

The *B. amyloliquefaciens* strain NJN-6 was isolated from the rhizosphere of a healthy banana plant in a FWB-suppressive soil. Field plots with plants pre-treated (in nursery pots) with a bio-organic fertilizer colonized by NJN-6 showed a decreased disease incidence by 68.5%, resulting in doubled yield (Xue et al., 2015). The mode of action of this strain relies on several metabolites. The lipopeptide iturin A, a powerful antifungal surfactant, is produced by several *Bacillus* strains including NJN-6 (Yuan et al., 2011). Two homologs of bacillomycin D and three homologs of members of the macrolactin family were identified in NJN-6 using HPLC/electrospray ionization mass spectrometry. Bacillomycin D and macrolactin exhibited significant antagonistic effects against *Foc* and *R. solanacearum*, respectively (Yuan et al., 2012a). Finally, among 36 VOCs detected in NJN-6, 11 compounds completely inhibited the *Foc* growth (Yuan et al., 2012b). Wang et al. (2013) isolated 57 bacterial strains from the rhizosphere of healthy banana plants grown in a field severely affected by the disease, all showing antagonism against *Foc*. Six strains (W2, W10, W14, W15, W17, and W19) displaying the best survival abilities in the rhizosphere soil were tested in greenhouse experiments, *B. amyloliquefaciens* W19 being the most effective against FWB. Moreover, and even more suggestive, biocontrol effectiveness of a bio-organic fertilizer colonized by W19 was then proved in a naturally infested field, where it reduced FWB by 44% and increased yield by 35% (Wang et al., 2016). Similarly to *B. amyloliquefaciens* NJN-6, the strain W19 produces several antifungal metabolites, including lipopeptides (e.g., iturin, bacillomycin D, and surfactin), 18 VOCs (Wang et al., 2013), and indole-3-acetic acid (Wang et al., 2016). Interestingly, banana root exudates seemed to enhance the ability of this strain to colonize roots by augmenting bacteria biofilm formation, due to surfactin production (Wang et al., 2016).

Munimbazi and Bullerman (1998) reported on extracellular antifungal metabolites produced by *B. pumilus* which inhibited the mycelial growth of different strains of *Aspergillus* sp., *Paenibacillus* sp., and *Fusarium* sp. The chitinolytic *B. pumilus* strain CH4 caused inhibition of *Foc* mycelial growth under *in vitro* conditions, but its effectiveness *in planta* has not been tested yet (Nawangsih and Purba, 2013).

The interest of *B. thuringiensis* as a BCA has been mainly focused on the Cry protein and its effect as bio-insecticide, a topic outside the scope of this review (Bravo et al., 2013, 2017). Nevertheless, it has also potential in biocontrol of phytopathogenic fungi due to the chitinase production. In fact, two chitinolytic strains of *B. thuringiensis* (50E and 48F) caused complete growth inhibition of *Foc* R4 *in vitro*. Indeed, uneven thickening and swelling of hyphae tips were observed at the sites where interaction with the bacterial crude chitinase took place (Usharani and Gowda, 2011).

### *Pseudomonas* spp.: The Metabolically-Versatile Biological Control Agents

*Pseudomonas* is a genus that comprises more than a hundred species (Mulet et al., 2010; Loper et al., 2012; Hesse et al., 2018). Many *Pseudomonas* spp. strains are indigenous inhabitants of the plant endosphere, rhizosphere, and/or phyllosphere, mostly established in these niches as commensals. Some of them are able to suppress the deleterious effects caused by different phytopathogens thereby promoting plant growth and health, and have thus been successfully used as plant inoculants (Arshad and Frankenberger, 1997; Haas and Défago, 2005; Mercado-Blanco and Bakker, 2007; Lugtenberg and Kamilova, 2009; Pliego et al., 2011; Schreiter et al., 2018). These bacteria displayed characteristics such as: (i) high colonization competence for plant surface, internal plant tissues (endophytism), and/or phytopathogen structures; (ii) versatility in the production of antibiotics suppressing diverse phytopathogens; (iii) ability to use specific nutrients in the target niche like plant exudates, that enable them to outcompete many components of the plant-associated microbiota; and (iv) capability to trigger (systemic) defense responses in the host plants (Mercado-Blanco, 2015, and references therein).

A relatively large number of *Pseudomonas* spp. strains have been studied as antagonists of *Foc*, the majority of which has been focused on species of the *fluorescens* group. A screening for the antagonistic activity of fluorescent pseudomonads isolated from the rhizoplane of several crops led to the identification of *P. fluorescens* Pf1 (Vidhyasekaran and Muthamilan, 1995). Since then, this strain has been studied, and proved effective, on several plant diseases, such as Fusarium wilt of chickpea (*F. oxysporum* f. sp. *ciceris*) (Vidhyasekaran and Muthamilan, 1995), rice blast (*Pyricularia oryzae*) (Vidhyasekaran et al., 1997), and rice sheath blight (*Rhizoctonia solani*) (Vidhyasekaran and Muthamilan, 1999; Nandakumar et al., 2001). *Pseudomonas fluorescens* Pf1 produces siderophores, hydrogen cyanide, the antibiotics 2,4-diacetylphloroglucinol (DAPG) and pyoluteorin, and induces resistance-associated enzymes (e.g., PO and PPO) in banana roots (Akila et al., 2011; Selvaraj et al., 2014). Repeated field trials have also demonstrated the effectiveness of *P. fluorescens* Pf1 against FWB, using diverse application protocols and formulations. Carriers such as wheat bran-saw dust (Raguchander et al., 1997), paring and pralinage, capsule (Raguchander et al., 2000; Rajappan et al., 2002), and talc (Rajappan et al., 2002; Saravanan et al., 2004b) have been explored. However, it is known that liquid formulations of *P. fluorescens* offer numerous advantages over solid formulations, e.g., high cell count, zero contamination, longer shelf life, greater protection against environmental stresses, and increased field efficacy (Hegde, 2002). Different chemicals, such as trehalose, polyvinylpyrrolidone, and glycerol, were tested for the development of a liquid formulation, and glycerol supported the highest Pf1 survival until 6 months of storage (Manikandan et al., 2010). A liquid formulation of Pf1 provided good results against FWB in multiple trials and locations (Selvaraj et al., 2014).

Saravanan et al. (2004a) reported a significant *in vitro* inhibitory effect on *Foc* R1 when testing five different *P. fluorescens* strains (Pf1, Pf2, Pf3, Pf4, and Pfm) isolated from banana rhizosphere, with the strain Pfm showing the highest antagonist effect against the pathogen growth. When greenhouse experiments were conducted using a talc-based formulation of strain Pfm, a significant reduction in vascular discoloration of the banana rhizome was observed (Saravanan et al., 2004a). Additionally, *P. fluorescens* Pfm systemically induced the accumulation of three key defense enzymes (PAL, PO, and polyphenol oxidase or PPO) in roots that contributed to induce resistance against *Foc* (Saravanan et al., 2004b).

Sivamani and Gnanamanickam (1988) investigated the possibility of suppressing FWB by bacterization with different *P. fluorescens* strains originating from roots of rice (strain Pfrl3), peanut (Pfgn), banana (Pfb), black gram leaves (Pfbg), citrus (Pfcp), and cotton (Pfco). These strains were tested for their *in vitro* antagonism ability against *Foc* R1 and R4. Results showed that strain Pfcp showed maximum inhibition of *Foc* mycelial growth, and was chosen to bacterize seedlings of *Musa balbisiana*. Seedlings treated with Pfcp showed less severe wilting symptoms and internal discoloration under greenhouse conditions. In addition, they also showed enhanced root growth and overall plant height.

In another study, 11 strains of *P. fluorescens* isolated from the banana rhizosphere were tested for their *in vitro* antagonistic effect against *Foc*. Among the tested isolates, strain Pf10 was the most effective in inhibiting the pathogen mycelial growth (Thangavelu et al., 2001). Further studies were focused on the effect of strain Pf10 treatment and *Foc* inoculation on the induction of banana plant enzymes and compounds known to be related with defense responses (e.g., PAL, POX, chitinase, β-1,3 glucanase and phenolics) (Thangavelu et al., 2003).

*In vitro* growth inhibition of *Foc* was also observed with *P. fluorescens* strain IIHRPf12. In greenhouse experiments using banana cv. Neeypovan, this strain reduced *Foc* colonization and FWB severity symptoms. Interestingly, structural modifications in the cortical cells at the site of fungal entry were observed, indicating that bacterized root cells were somehow “alerted” to mobilize a number of defense structures aiming to hinder the pathogen progress (Mohandas et al., 2004).

Goswami et al. (2015) reported on the *P. aeruginosa* strain BG, isolated from marine water from the Gulf of Khambhat in Gujarat, and its ability to inhibit *Foc* growth under *in vitro* conditions. *Pseudomonas aeruginosa* BG displayed plant growth promotion, biocontrol abilities, secretion of enzymes such as catalase, urease, and phosphatase, as well as the synthesis of metabolites such indole-3-acetic acid, siderophores, ammonia, and hydrogen cyanide. Other *P. aeruginosa* were also tested against *Foc in vitro* (Ayyadurai et al., 2006; Sekhar and Pious, 2015) and *in planta* (Yu C. et al., 2010).

### *Trichoderma* spp.: Antagonists and Plant Growth Promoters

*Trichoderma* is a genus of asexually-reproducing fungi widely distributed in nearly all temperate and tropical soils. The sexual teleomorph (genus *Hypocrea*) can be found frequently, but many strains, including most biocontrol strains, have no known sexual stage. *Trichoderma* spp. show a wide genetic diversity, and are producers of several extracellular proteins, enzymes such as cellulase and chitinase, and more than 100 different metabolites with antibiotic activities. This genus can also parasitize a range of other fungi (e.g., *R. solani*). Besides antibiosis and mycoparasitism, *Trichoderma*-mediated biocontrol also relies on the induction of plant resistance (Harman et al., 2004). Therefore, due to their metabolite arsenal, rhizosphere-competence, and ability to stimulate plant growth, *Trichoderma* species have long been recognized as BCAs (Harman et al., 2004; Vinale et al., 2008; Woo et al., 2014), and they are widely studied against FWB (Supplemental Figure 2). These fungi are efficient colonizers of plant roots, where they establish intense interactions with plants (Vinale et al., 2008). They colonize root surfaces and invade the root epidermis, usually not beyond the first or second layer of cells (Yedidia et al., 2000), but some authors have claimed the endophytic nature of the strains tested in their research, even in banana (Caballero Hernández et al., 2013; Thangavelu and Gopi, 2015a; Chaves et al., 2016).

A rhizospheric strain, namely *T. viride* NRCB1, was identified *in vitro* among 37 isolates and tested in the pot- and field-experiments, where it reduced FWB by a maximum of 75–80% in terms of external symptoms and vascular browning (Thangavelu and Mustaffa, 2010). The authors proved that a bioformulation based on the rice chaffy grains conferred a higher efficacy over the talc cum powder formulation. Finally, the strain was able to induce the peroxidase (PO), phenylalanine ammonia lyase (PAL), and total phenolic content in treated plants (Thangavelu and Mustaffa, 2010). Later, the same authors identified a new rhizospheric strain (*Trichoderma* sp. NRCB3) which was combined with the endophyte *Trichoderma asperellum* Prr2, and successfully tested against FWB in the field (Thangavelu and Gopi, 2015a). Significant disease protection was obtained in the field using a *T. viride* strain applied by root dipping at transplanting and by a colonized wheat bran:saw dust mixture 3 months later. The treatment reduced FWB incidence by 75% and increased the yield by 60% (Raguchander et al., 1997). Interesting results were also obtained using a commercial product namely ECO-T® (containing *T. harzianum*; Plant Health Products, South Africa), though they were context-specific. In fact, the product reduced by 68% the *Foc* inoculum in Humic Nitisol, but only by 6% in Rhodic Ferralsol (Mukhongo et al., 2015). With the assumption that potential microbial antagonists are more abundant in soil with a history of low disease incidence, Nel et al. (2006) selected two *Trichoderma* spp. isolates, T22 and T5, from FWB-suppressive soil in South Africa, and obtained with them a 62% FWB control in a glasshouse (*Foc* R4). These strains were among the most effective strains tested in the experiment (33 strains) and were superior to a *Trichoderma*-based commercial product. Interesting results (efficacy higher than 80%) were also obtained on abaca (*Musa textilis*) and plantain (Bastasa and Baliad, 2005; Pérez Vicente et al., 2009). The literature, however, also reports examples of failure in the control of *Foc* (R4) using *Trichoderma* spp. strains, albeit resulting effective *in vitro* or able to delay the disease onset (Ting et al., 2003; Wibowo et al., 2013).

### Arbuscular Mycorrhizal Fungi: Not Only Nutrient Providers for the Plants

The root cortical tissues enable the host plant to live in a symbiotic association (intercellularly or intracellularly) with fungi of the phylum Glomeromycota, which develop morphological structures called arbuscules and transform the infected root into the so-called “mycorrhiza.” These arbuscular mycorrhizal fungi (AMF) gain some nutrients from the plant and return others to it, thus enhancing the plant growth. Additionally, AMF protect plants against phytopathogens and abiotic stresses (Parniske, 2008; Bonfante and Genre, 2010; Lenoir et al., 2016). The AMF's beneficial effects have been also demonstrated in banana, albeit sometimes with inconstant results. While AMF are generally considered as plant growth promoters rather than pathogen antagonists, it is fascinating to see how AMF inoculated in the banana nursery could provide, in some cases, protection from FWB in the field during several subsequent weeks. Indeed, an early study reported that either *Glomus intraradices* or *Glomus* spp. stimulated the growth of banana plants cv. Grande Naine and reduced both rhizome necrosis and external FWB symptoms (Jaizme-Vega et al., 1998). Inoculation with *Gigaspora margarita* was found to reduce FWB in pot-grown plants cv. Maçã, especially under low concentration of *Foc* inoculum (Borges et al., 2007). Also, the pathogen population in roots of banana plants at 7 months after treatment with a combination of *G. mosseae* and *T. harzianum* was significantly reduced, as measured by ELISA (Mohandas et al., 2010). In the field, banana plants pre-treated in the nursery with *G. clarum* had higher biomass than untreated plants, and showed lower FWB incidence (67%), compared to the untreated control (88%). But plants pre-treated with *G. clarum* and then inoculated at transplanting with a commercial product based on *P. putida* and *T. asperellum* did not differ from the untreated control in FWB incidence and severity (Lin et al., 2012). The lack of knowledge about the interactions among those BCAs might have led to unexpected results.

It is known that plant colonization by AMF is stimulated by the soil organic matter and hindered by mineral fertilization. In banana cv. Maçã, the application of a bio-fertilizer promoted abundant mycorrhizal colonization and was associated to lower expression of FWB symptoms, compared to applications of the Hoagland solution at three concentrations (non-fertilized control was not established) (Sampaio et al., 2012). However, inconsistent results obtained with AMF have been a common experience. In a factorial experiment, the effectiveness of a commercial AMF-based product and two other commercial BCAs was context-specific. Three products were inoculated in the nursery and their efficacy against FWB evaluated on “Gros Michel” banana grown in fields with three different soil types. Rhizatech® (Dudutech, Kenya) reduced *Foc* inoculum by 55% in Humic Nitisol. In this soil type, PHC Biopak® (*Bacillus* spp.; Plant Health Care Inc., USA) reduced *Foc* by 47%, a level similarly reached in Vertisol (50%). ECO-T® (*T. harzianum*; Plant Health Products, South Africa), also showed the best efficacy in Humic Nitisol (68% disease control), while it was almost ineffective in Rhodic Ferralsol (6%) (Mukhongo et al., 2015).

### Non-pathogenic *Fusarium oxysporum*: Beneficial Relatives

The species *F. oxysporum* includes pathogenic strains as well as plant beneficial endophytes and saprophytes living in soil and on organic debris (Di Pietro et al., 2003). Non-pathogenic *F. oxysporum* (np*Fo*) strains are primarily recognized upon their inability to infect plants. Since about 120 *formae speciales* are known for *F. oxysporum*, np*Fo* should be validated on as many species by pathogenicity tests. Nevertheless, it is generally accepted that a limited number of plant species is enough to declare an *F. oxysporum* as np*Fo* (Fravel et al., 2003). Determining the vegetative compatibility group (VCG) cannot be used as a universal tool to identify np*Fo* isolates. Nel et al. (2006) developed a PCR-based restriction fragment length polymorphism (RFLP) analysis of the rRNA intergenic spacer (IGS) region for discriminating np*Fo* strains from *Foc* among 100 isolates obtained from banana rhizosphere in South Africa. The mechanisms underlying the biocontrol exerted by np*Fo* strains are based on the competition for infection sites or nutrients, as well as the induction of systemic resistance (He C. Y. et al., 2002; Fravel et al., 2003; Olivain et al., 2006). The fact that np*Fo* share the same niche with the pathogen is advantageous from the biocontrol perspective (Larkin and Fravel, 2002).

Two np*Fo* namely Ro-3 and Ra-1 reduced FWB (*Foc* R1) by 80% on cv. “Rasthali” in a field trial. They were selected *in vitro*, proved to be effective in reducing the disease (up to 89% reduced severity) and promoting the plant growth when applied three times on both tissue-cultured and sucker-derived plants under greenhouse conditions (Thangavelu and Jayanthi, 2009). Another np*Fo* isolate, UPM31P1, alone or in combination with *S. marcescens* isolate UPM39B3, resulted effective *in vitro* against *Foc* TR4, and reduced FWB under greenhouse condition. In the field, this strain only delayed the onset of FWB (*Foc* TR4), as it reduced the percentage of diseased plants by 75% at 15 weeks post-transplanting, but no significant difference between treated and untreated plants was observed at 28 weeks post-transplanting (Ting et al., 2009c). Plants treated with two np*Fo* isolates obtained from disease suppressive soils in South Africa, CAV 255 and CAV 241, showed a FWB incidence reduced by 87.4 and 75.0%, respectively (Belgrove et al., 2011). In the same trial, the widely studied strain Fo47 did not suppress significantly the disease. Forsyth et al. (2006) pointed out that np*Fo* isolates may unexpectedly be synergistic with *Foc*, thus they are not necessarily antagonists. In fact, although more evidence would be needed, the strain BRIP 45952 increased Fusarium wilt disease severity on “Cavendish.” Nevertheless, another isolate, BRIP 29089, reduced disease severity in artificially inoculated “Lady Finger” (*Foc* R1) and “Cavendish” (*Foc* STR4) plants. At least three more research works have dealt with np*Fo* isolated from FWB suppressive soil and/or banana plants, though their assays remained at the laboratory stage (Nita and Harsh, 2015) or were not related to biocontrol (Nel et al., 2006; Deltour et al., 2018).

### *Streptomyces* spp. and Other Actinomycetes: Natural Antibiotics Factories

Streptomycetes are the most important antibiotic-producing microbes. They also produce a broad range of additional secondary metabolites and lytic enzymes. For this reason, they receive attention for biotechnological, pharmaceutical and agricultural purposes. Streptomycetes are widely distributed in the soil, where they are strong competitors and antagonists. The use of streptomycetes as BCAs is largely documented in the literature and, recently, their application against plant diseases incited by *Fusarium* species has been reviewed by Bubici (2018). In the literature, we found only four pot trials where streptomycetes were tested against FWB (Table 1). All four experiments were conducted against *Foc* R4 and showed that FWB could be reduced between 46 and 87%. Interestingly, the highest disease reductions were obtained using the streptomycete fermentation broth (Qin et al., 2010; Zhou et al., 2017), compared to the experiments where spore suspensions were applied by drenching or root dipping (Cao et al., 2005; Getha et al., 2005). Qin et al. (2010) selected 8 out of 139 isolates using *in vitro* assays against several *F. oxysporum formae speciales* and demonstrated that the application of their fermentation broth provided a FWB control ranging from 78 to 87% in pot experiments. In particular, using 1.85·10^6^ conidia mL^−1^ of *Foc* R4, plants treated with the best streptomycete strain, ZJ-E1-2, showed FWB incidence of 12%, while it was 76% on untreated trees. With a similar disease pressure, *viz*. 78% incidence on the control plants upon inoculation with 1·10^6^ CFU mL^−1^ of *Foc* R4, Zhou et al. (2017) observed a FWB reduction of 73% after treatment with *S. lunalinharesii* B-03. The application of fermentation broth introduces into the soil both the microbial cells and their metabolites and, hence, it has a stronger impact on soil *Foc* inoculum than the sole microbial cells. In fact, when introduced alone, the cells must first proliferate to produce antifungal metabolites enough for effective control of *Foc*. Trees treated before planting with 10^6^ CFU mL^−1^ of *Streptomyces* sp. strain S96, and later inoculated with 10^4^ conidia mL^−1^ of *Foc* R4, showed significant reductions in FWB incidence, severity, and vascular browning. *Streptomyces* sp. strain S96 was selected from 131 endophytic actinomycetes isolated from surface-sterilized banana roots (Cao et al., 2005). The soil application of a spore suspension (10^8^ CFU mL^−1^) of *Streptomyces* sp. strain g10 reduced FWB severity index by 47% and rhizome discoloration by 53% when banana plantlets were inoculated with 10^4^ conidia mL^−1^ of *Foc* R4. The same treatment was ineffective under higher pathogen pressure, i.e., 10^6^ conidia mL^−1^ (Getha et al., 2005). *Streptomyces* sp. strain g10 was effective *in vitro* against several phytopathogenic fungi, including different physiological races of *Foc* (Getha and Vikineswary, 2002). Nevertheless, *Foc* and *R. solani* were more resistant than other fungi (i.e., *P. oryzae* and *Phytophthora palmivora*) to the antagonistic streptomycete (Getha et al., 2004). Crude fractions containing antifungal metabolites excreted in liquid media by g10 produced swelling, distortion and excessive branching of *Foc* R4 hyphae, as well as inhibition of spore germination. Antibiosis-mediated *Foc* antagonism was also demonstrated in sterile soils for the strain g10 by using an indirect method, i.e., the paper disc method (Gunji et al., 1983).

Several articles reported on *in vitro* experiments with *Streptomyces* spp. strains, but assays under *in vivo* conditions to fully demonstrate their biocontrol effectiveness have not made yet (Table 2). In these studies new antifungal metabolites were discovered, such as (6S,8aS,9S,11S,12aR)-6-hydroxy-9,10-dimethyldecahydrobenzo[d]azecine-2,4,12(3H)-trione (termed as 210-A) (Wu et al., 2009), and fungichromin (Wei et al., 2011). Other three compounds were isolated from *S. albospinus* 15-4-2: 2-methyl-2,5,6-bornantriol, 4,4′-(3-hydroxypropane-1,1-diyl)diphenol, and 7-(4-methoxybenzyl)-4,5,6,7-tetrahydro-1,3-oxazepine-5,6-diol. These compounds did not show an inhibitory effect against *Foc* R4, though the streptomycete was effective against the same pathogen (Yu et al., 2011). Two other studies demonstrated the efficacy of crude culture filtrate or methanol extracts of streptomycetes, but the effective metabolites were not identified (Shih et al., 2013; Wang L. et al., 2015). Soil inoculation with *S. griseus* St 4 viable cells was more effective in suppressing *Foc* TR4 (6 log_10_ CFU g^−1^ soil of *Foc*) than cell-free crude extracts (7 log_10_ CFU g^−1^ soil) at 20 days after inoculation (Zacky and Ting, 2013). The formulation of *S. griseus* St 4 with kaolin clay, sodium alginate, or a kaolin-alginate combination increased the effectiveness of the streptomycete, compared to non-formulated cells. The kaolin clay formulation reduced *Foc* TR4 soil inoculum from 6 to 5.4 log_10_ CFU g^−1^ soil (Zacky and Ting, 2015).

### Other Genera or Unidentified Species of Biological Control Agents

A *Serratia marcescens* strain, isolated from roots of wild bananas, has shown plant growth promoting effect both in glasshouse and field, and suppressed FWB, though only in the glasshouse. The loss of control efficacy stimulated the evaluation of diverse formulations in an attempt to improve its viability and efficacy in field applications. Results showed that bentonite performed better and that further advantage could come from optimization of non-fat skimmed milk and sucrose levels, whereas para-aminobenzoic acid should be omitted from bentonite formulations (Ting et al., 2009a; Ting A.S.Y. et al., 2011). Other studies in greenhouse evaluating several rhizospheric bacterial strains (FB5, FB2, T2WF, T2WC, and W10) of unidentified species culminated in the successful control of FWB (81% reduction) (Yang et al., 2006). Moderate control (16%) was obtained using *Paenibacillus* spp. strains RZ-17 and RZ-24 which also had additional effects on mortality and motility of *Meloidogyne javanica* second stage juveniles (Ribeiro et al., 2012). Multiple beneficial effects have been also reported for strains of marine rhizobacteria isolated from mangrove (YS4B1, YS1A3, and YS2A5), which were also effective against *R. solanacearum* and *Mycosphaerella fijiensis* (Bonsubre et al., 2016). Finally, a number of studies identified *Foc* antagonists among strains of *Talaromyces* spp., *Eutypella* sp., *Paenibacillus polymyxa, Herbaspirillum* spp., *Tsukamurella paurometabola, Brevibacillus brevis*, and *Streptoverticillium lavenduligriseum* (Sun et al., 2010; Manoch and Dethoup, 2011; Ting S. et al., 2011; Marín et al., 2013; Shen L. et al., 2013; Sun and Hsieh, 2015; Qi et al., 2017). A spectacular control of FWB was claimed by Chand et al. (2016), who applied a dead *Foc* to plant roots before inoculation with live *Foc*. Although no data were showed, the authors stated that inoculated plants, grown in a sick plot, did not show disease symptoms even 2 months after inoculation, while they occurred on control plants 1 week after inoculation. This approach appears as interesting as it is unusual, and no other researchers have replicated the technique against *Foc* until now.

## Fusarium Wilt of Banana and the Banana-Associated Microbiomes

The development of next-generation sequencing (NGS) technologies, along with advanced bioinformatics tools, is rapidly increasing our knowledge on many biological processes. Diverse “-omics” techniques such as (meta)genomics, (meta)transcriptomics, proteomics, metabolomics, microbiomics, etc., are currently available to better understand plant-microbe(s) interactions from a holistic perspective (Massart et al., 2015). However, the implementation of “-omics” in the study of BCAs effective against FWB as well as their interaction with the banana-*Foc* pathosystem is still very scant.

The NGS-based approaches are very useful for the in-depth study of the structure, composition, and diversity of plant-associated microbiomes. Yet, microbiomics is waiting to be used in a more frequent way in the research field of banana, FWB, and biocontrol. Recent studies (*16S rRNA* and *ITS* amplicon sequencing profiling) have been focused on endophytic bacterial communities present in different parts and micro-environments of banana plants (Köberl et al., 2015, 2017; Zhai et al., 2016; Suhaimi et al., 2017), as well as in the microbiota of banana rhizosphere (Fu et al., 2016a) and soil (Xue et al., 2015; Rames et al., 2018).

Healthy plants and healthy soils have higher microbial diversity and more abundant beneficial microbes, which can improve nutrients uptake, promote plant growth, and control soil-borne diseases (Bulluck and Ristaino, 2002; Bailey and Lazarovits, 2003; Raaijmakers et al., 2009; Luan et al., 2015). Köberl et al. (2015) studied the impact of biogeography and agroforestry on the banana-associated microbiome, mostly γ-proteobacteria. Banana plants grown under agroforestry systems showed a higher abundance of potentially beneficial plant-associated bacteria and lower presence of phytopathogenic bacteria. Thus, γ-proteobacteria diversity and community members were identified as potential health indicators. Healthy plants revealed an increase in potentially beneficial microbes like *Pseudomonas* and *Stenotrophomonas*, while diseased plants showed a preferential occurrence of *Enterobacteriaceae* (Köberl et al., 2017). Another study correlated FWB positively with the abundance of *Proteobacteria, Ascomycota, Fusarium, Cylindrocarpon, Gymnascella, Monographella, Pochonia*, and *Sakaguchia*, but negatively with *Acidobacteria, Firmicutes, Leptosphaeria*, and *Phaeosphaeriopsis* (Shen et al., 2015a). In such pot-experiment, 2 years of biofertilizer application manipulated the composition of the rhizosphere microbial community and induced the FWB suppression. The relationship among suppression of *Foc* under field conditions, the use of ground cover management, and changes in the soil microbiome was also investigated in “Ducasse” banana (synonym “Pisang Awak,” ABB) (Pattison et al., 2014; Rames et al., 2018). Results showed that suppression of FWB tended to increase over time when the banana was cultivated with ground covers compared to bare soil conditions. Statistically significant changes over time in the structure of soil microbial communities in the vegetated treatment were observed, and potential biomarkers related to disease suppression were identified. In addition, fungal amplicon sequencing demonstrated that reduction of *Foc* in the vegetated treatment was associated with disease suppression.

Similarly, analyzing the banana/*R. solanacearum* pathosystem (bacterial wilt of banana or Moko), five major microbial genera were found in both symptomatic and non-symptomatic plant samples: *Sphingomonas, Methylobacterium, Flavobacterium, Pseudomonas*, and *Ralstonia*, the latter being more abundant in symptomatic (59% out of the entire genera) than in non-symptomatic plants (only 36%). In addition, several genera were only assigned to non-symptomatic plants (Suhaimi et al., 2017). Another experiment showed that the soil cultivated with tobacco and infested by *R. solanacearum* had lower microbial diversity than the soil free from the pathogen, which harbored more abundant beneficial microbes such as *Bacillus, Agromyces, Micromonospora, Pseudonocardia, Acremonium, Lysobacter, Mesorhizobium, Microvirga, Bradyrhizobium, Acremonium*, and *Chaetomium*. Also, the activities of catalase, invertase, and urease, as well as soil pH, available phosphorus and potassium content were lower in the infested soils (Wang et al., 2017).

Soil microbial community varies because of many factors. The main drivers of the rhizosphere microbiome are soil type and plant genotype (Berg and Smalla, 2009), but fertilizers (Ikeda et al., 2011), crop rotation (Hilton et al., 2013), and pesticides (Jacobsen and Hjelmso, 2014) may also play a significant role. Soil microbiota also fluctuates with the plant growth stages, mostly due to changes in the root exudates (Yang and Crowley, 2000; Okubo et al., 2016; Wang et al., 2017). Finally, the soil microbial assembly may be influenced by BCAs artificially introduced in the system (Xue et al., 2015). However, several studies have revealed that edaphic and anthropic factors had a deeper and more durable effect on the rhizosphere microbiota than a BCA application. For example, compared to untreated plants, *R. solani* had a much higher impact on lettuce rhizosphere bacterial communities than the applications of diverse BCAs such as *Trichoderma* sp. (Grosch et al., 2006), *P. jessenii* RU47 (Adesina et al., 2009), or *B. amyloliquefaciens* FZB42 (Chowdhury et al., 2013; Erlacher et al., 2014). On the other hand, co-inoculation of different BCAs may cause a more pronounced impact on the microbial community structure compared to the single strain application, as demonstrated in the lettuce rhizosphere (Grosch et al., 2012). Nonetheless, some systems may be more reluctant to changes, sometimes even contrasting the applied BCAs (Garbelotto et al., 2019).

The “-omics” technologies do not only provide a global overview of the banana-associated microbiota but may also yield useful information to develop more effective biological control strategies. For instance, dominant bacterial groups can be identified in FWB suppressive soils, thereby leading to the development of strains or consortia of strains serving as new biocontrol tools. For example, the above mentioned *B. amyloliquefaciens* strain NJN-6 was isolated from a FWB-suppressive soil after the NGS analysis had evidenced *Bacillus* as the dominant taxon (Xue et al., 2015). A metagenomic study has been targeted to microbes that harbor the non-ribosomal peptide-synthetase (NRPS) gene, which encodes for one of the largest groups of natural microbial secondary metabolites, such as the antibiotics vancomycin and gramicidin, as well as the lipopeptides surfactin, iturin A, and bacillomycin. The research evidenced that these microbes were more abundant in FWB-suppressive soil than in FWB-conducive soil. The main microbial taxa harboring the NRPS gene and related to FWB suppression were *Pseudomonas* spp. and *Streptomyces* spp. (Zhao et al., 2018). As potential probiotic candidates, plant vertically transmitted actinobacteria are beneficial to the growth and health of host plants (Du et al., 2018). The majority of bacteria from healthy banana shoot tips were affiliated with actinobacteria, being *Mycobacterium* and *Nocardia* the dominant taxa. The streptomycetes were isolated from shoot tips and proved to enhance the growth and resistance to *Foc* of pot-grown banana plants. The research elegantly presented how microbiomics can foster the selection for probiotic agents (Du et al., 2018). Another study has shown that the endophytic root microbiome of healthy banana plants was dominated by Nocardioidaceae (56.37%), Pseudonocardiaceae (14.36%) and Nocardiaceae (9.77%) (Zhai et al., 2016).

But metagenomics and microbiomics are not the sole “-omics” technologies that can help the research on biocontrol of FWB. Nevertheless, the implementation of “-omics” techniques other than microbiomics for studying biocontrol mechanisms of *Foc* is still absent or very limited. The study of a tripartite interaction among banana, *Foc*, and *T. asperellum* strain Prr2 is one of the few examples available, however with a non-NGS approach, *viz*. the suppression subtractive hybridization or SSH (Thangavelu et al., 2016).

## Gaps in the Knowledge and Concluding Remarks

Banana production systems lack phytosanitary certification schemes. In Australia, where *Foc* TR4 is considered a quarantine pest, the organization that governs standardization and certification of agricultural practices, GlobalG.A.P., launched in 2017 an “add-on” in its standards which encourages farmers to take preventive biosecurity measures following a strict protocol (GLOBALG.A.P, 2019). However, it is only a preventive awareness measure rather than a true certification. Phytosanitary certification would be necessary either for tissue culture- or sucker-derived plants, because *Foc*-contamination may occur at different stages of plant production. In fact, undesired infected plants can derive not only from infected asymptomatic suckers but also from pathogen-free tissue culture plantlets, which can be later contaminated by the pathogen during the acclimation period (e.g., by contaminated irrigation water or substrate).

It is worth noting that, despite its dangerousness, *Foc* is not yet a quarantine pathogen in several countries, and where it is considered as such, the regulation is limited to TR4 only. For example, in countries joining to the European and Mediterranean Plant Protection Organization (EPPO), *Foc* is not yet reported in the A1 or A2 lists, which include pests and pathogens absent or present, respectively, in the EPPO region and recommended for quarantine regulations (EPPO, 2019). Among the *Fusarium* species, only *F. circinatum, F. euwallaceae, F. foetens* and *F. oxysporum* f. sp. *albedinis* are included in the A2 list, while *F. oxysporum* f. sp. *lactucae* is provisionally placed in an alert list because it is pending for a risk assessment that will designate or not it as a quarantine pathogen. Therefore, in the EPPO region, no evaluation for the inclusion of *Foc* among the quarantine pathogens seems to have initiated yet. Nevertheless, awareness campaigns, pest risk assessments, and research works have largely emphasized the importance of quarantine measures against *Foc* TR4 (Baker et al., 2008; Pocasangre et al., 2011; Blomme et al., 2013; Sánchez, 2013; Anses, 2018). Both quarantine measures and phytosanitary certification schemes require huge efforts by the governments in terms of legal framework, personnel training, and protocols for inspection, sampling, diagnosis, etc.

Following the pathogen exclusion and quarantine measures, which are critical to hinder the current *Foc* TR4 expansion, the use of pathogen-free certified plating material is certainly one of the first key preventive steps toward successful management of FWB. It has been implementing well in countries of Latin America, where almost 100% of plants derived from tissue culture, but only partially in Africa, where suckers are still largely used to establish new (especially small) plantations, very likely for economic and cultural reasons. On the other hand, the introduction of beneficial and well-characterized microorganisms during banana propagation protocols can be an excellent strategy to “prepare” (or “pre-condition,” or “prime”) plantlets to cope with *Foc* inoculum in the field.

A large number of studies have contributed to select BCAs with variable effectiveness against *Foc*, and many of them reached the field-testing stage (Table 1 and Supplemental Figure 2). Interestingly, the disease control degree obtained experimentally has been surprisingly high, even considering that *Foc* is a vascular pathogen enduring in the soil for a long time and, thus, it is generally considered difficult to control using BCAs. Bio-formulation, which is one of the key factors affecting the BCA efficacy, has been tested in several cases, but new aspects, such as nanotechnology and formulation of microbial consortia and/or their metabolites (Keswani et al., 2016), merit to be studied. Moreover, the effectiveness of these formulations could be improved by the amendment with specific nutrients/factors aiming to enhance the biosynthesis of these metabolites as well as the survival of the BCAs in the banana rhizosphere. Microbial metabolites have proved their value in nutrition, agriculture, and healthcare, but poorly evaluated so far against FWB. In contrast, plant extracts like *A. tuberosum* (Figure 3) have been repeatedly tested, especially during the last years (Singh et al., 2017). Bioformulations combining different strains, nutrients, metabolites and/or other natural products must take into account the compatibility (i.e., the absence of antagonism) of the different components (Sarma et al., 2015; Gómez-Lama Cabanás et al., 2018). Blends of microorganisms beyond their mere mixture, but based on tailor-made combinations of strains with complementary and/or synergistic modes of action are encouraged (Lutz et al., 2004). Solidly supported by the powerful currently-available “-omics” methodologies, biocontrol strategies can now aim to provide novel tools based on *ad hoc* tailored consortia of BCAs originating from the indigenous microbiota associated with the target plant (Gopal et al., 2013; Berg et al., 2017; Mercado-Blanco et al., 2018), thereby overcoming problems and inconsistencies frequently observed when biopesticides are based on formulations of a single microorganism or combinations of few of them. Furthermore, successful colonization and endurance in the target niche must be guaranteed as the first requisite for successful biocontrol. Therefore, a comprehensive understanding of the mode of action, ecology, and trophic interactions established upon the application of BCAs is instrumental for their success (Saraf et al., 2014; Eljounaidi et al., 2016; Shafi et al., 2017). Monitoring the microbial strains in the field after their application should be addressed in order to understand their fate in the environment and optimize their application protocol. For these aspects, “-omics”-based approaches are of great help.

The availability of the *M. acuminata* draft genome marked a milestone in the genetic research of banana (D'Hont et al., 2012), and the genomic sequence of several *Foc* races is also available. Furthermore, transcriptomics has been implemented in studies on *Musa* spp. (e.g., Backiyarani et al., 2015) enhancing our knowledge on physiological processes such as the fruit ripening (Asif et al., 2014) and the responses to abiotic stresses such as low-temperature (Yang Q. S. et al., 2015). This “-omics” has been largely used to study the interaction between BCAs, plant and/or microbiome, but no studies are available on the banana/*Foc* pathosystem. Moreover, integrating genomics, metagenomics and (meta)transcriptomics would allow understanding the microbiota structure and the roles and functions of its members, as well as the intricate interactions between BCAs, microbiota, plant and the environment. This approach has not been applied to plant microbiomes yet, but it is providing new insight in other fields like the human microbiome (Massart et al., 2015). Proteomics and metabolomics analyses have been also conducted in banana, but they have been focused mainly on the plant-pathogen interaction while BCAs have not been involved (Li et al., 2013, 2017; Lu et al., 2013; Sun et al., 2014; Ramu et al., 2016; Gopalakrishnan, 2017; Yuan et al., 2017). Therefore, at least to the best of our knowledge, there is still an important gap in our knowledge of biocontrol against *Foc*, including insight on the mechanisms involved in the antagonism, plant colonization, plant growth promotion, etc.

Biocontrol should not be considered as an independent tool, but adequately implemented in an integrated management framework. Actually, besides the combination with organic fertilizers, very little has been investigated on the integration of BCAs with other control means. For instance, the effect of an organic amendment against FWB was enhanced by the combination with a BCA, *B. amyloliquefaciens* strain NJN-6, namely biofertilizer (Shen et al., 2015a). Furthermore, the biocontrol efficacy of such biofertilizer resulted even higher when it was applied after ammonia fumigation (Shen et al., 2019). Other studies have shown that combinations of biocontrol organisms with silicon and mulching, or with neem cake can be advantageous compared to the individual applications, and therefore can provide a better control option for banana growers who have to deal with FWB in their plantations (Saravanan et al., 2003; Kidane and Laing, 2010). Diverse combinations of treatments with silicon, *T. harzianum*, compost, various sources of nitrogen, phosphorus and potassium, and the cover crop *Crotalaria juncea* were applied in the field to banana varieties differing in the susceptibility level to *Foc*. The results highlighted the advantages of integrated disease management, especially the combination of different control means with the host genetic resistance. In fact, the treatment including all the control means was more effective than those including only some of them. Also, while in the highly susceptible cultivar Silk (AAB) the treatment was not effective in reducing FWB during the first crop cycle, in the moderately susceptible variety Prata Anã (AAB) it reduced the disease by 58%, with a yield increment of 157.3% (Haddad et al., 2018).

We showed that the literature offers numerous examples of encouraging results, suggesting that biocontrol can greatly contribute to limit the damage caused by FWB. More efforts should be done to further validate the currently available outcomes, to deepen the knowledge on the most valuable BCAs, and to improve their efficacy by setting up effective formulations, application protocols, and integrated strategies.

## Author Contributions

All the authors wrote sections of the manuscript, contributing equally to its first draft. GB coordinated and merged the individual contributions from the authors. All the authors read, revised, and approved the submitted version.

### Conflict of Interest Statement

The authors declare that the research was conducted in the absence of any commercial or financial relationships that could be construed as a potential conflict of interest.

## Supplementary Material

Supplemental Figure 1 Publication timeline of scientific articles dealing with Fusarium wilt of banana. Articles were retrieved from the CAB Direct database (1970–2018) by searching the keywords “*Fusarium cubense*” or “Panama disease” in the title and abstract (735 articles). *Foc*: *Fusarium oxysporum* f. sp. *cubense*; FWB: Fusarium wilt of banana.

Supplemental Figure 2 Biological control agents studied for the control of *Fusarium oxysporum* f. sp. *cubense*. The numbers indicate the scientific articles retrieved from CAB Direct database (1970–2018) by searching the keywords “*Fusarium cubense*” or “Panama disease” in the title and abstract.

Supplemental Table 1 List of *in vitro* experiments conducted against *Fusarium oxysporum* f. sp. *cubense* (*Foc*) using beneficial microorganisms.
