# Corrigendum: Biological Control Agents Against Fusarium Wilt of Banana

**DOI:** 10.3389/fmicb.2019.01290

**Published:** 2019-06-11

**Authors:** Giovanni Bubici, Manoj Kaushal, Maria Isabella Prigigallo, Carmen Gómez-Lama Cabanás, Jesús Mercado-Blanco

**Affiliations:** ^1^Consiglio Nazionale delle Ricerche (CNR), Istituto per la Protezione Sostenibile delle Piante (IPSP), Bari, Italy; ^2^International Institute of Tropical Agriculture (IITA), Dar es Salaam, Tanzania; ^3^Department of Crop Protection, Institute for Sustainable Agriculture (CSIC), Córdoba, Spain

**Keywords:** *Musa acuminata*, *Fusarium oxysporum* f. sp. *cubense*, Panama disease, soil microbiota, beneficial microorganisms, biocontrol

In the original article, the reference for “Kalaiponmani et al., 2017” was incorrectly written as “Gopalakrishnan, V. (2017). Optimization of protein isolation and preliminary comparative proteomics of pathogenic *Fusarium oxysporum* f. sp. *cubense* (p-Foc) and non-pathogenic Fusarium oxysporum (np-Fo). *J. Plant Pathol*. 99, 361–369. 10.4454/jpp.v99i2.3883”

It should be “Kalaiponmani, K., Thangavelu R., and Varun, G. (2017). Optimization of protein isolation and preliminary comparative proteomics of pathogenic *Fusarium oxysporum* f. sp. *cubense* (p-Foc) and non-pathogenic *Fusarium oxysporum* (np-Fo). *J. Plant Pathol*. 99, 361–369. 10.4454/jpp.v99i2.3883”

The authors apologize for this error and state that this does not change the scientific conclusions of the article in any way.

