# Supplementary figures and images for: Biological Control Agents Against Fusarium Wilt of Banana
Source: Front Microbiol. 2019 Apr 5;10:616. doi: 10.3389/fmicb.2019.00616 (PMC6459961; doi:10.3389/fmicb.2019.00616)

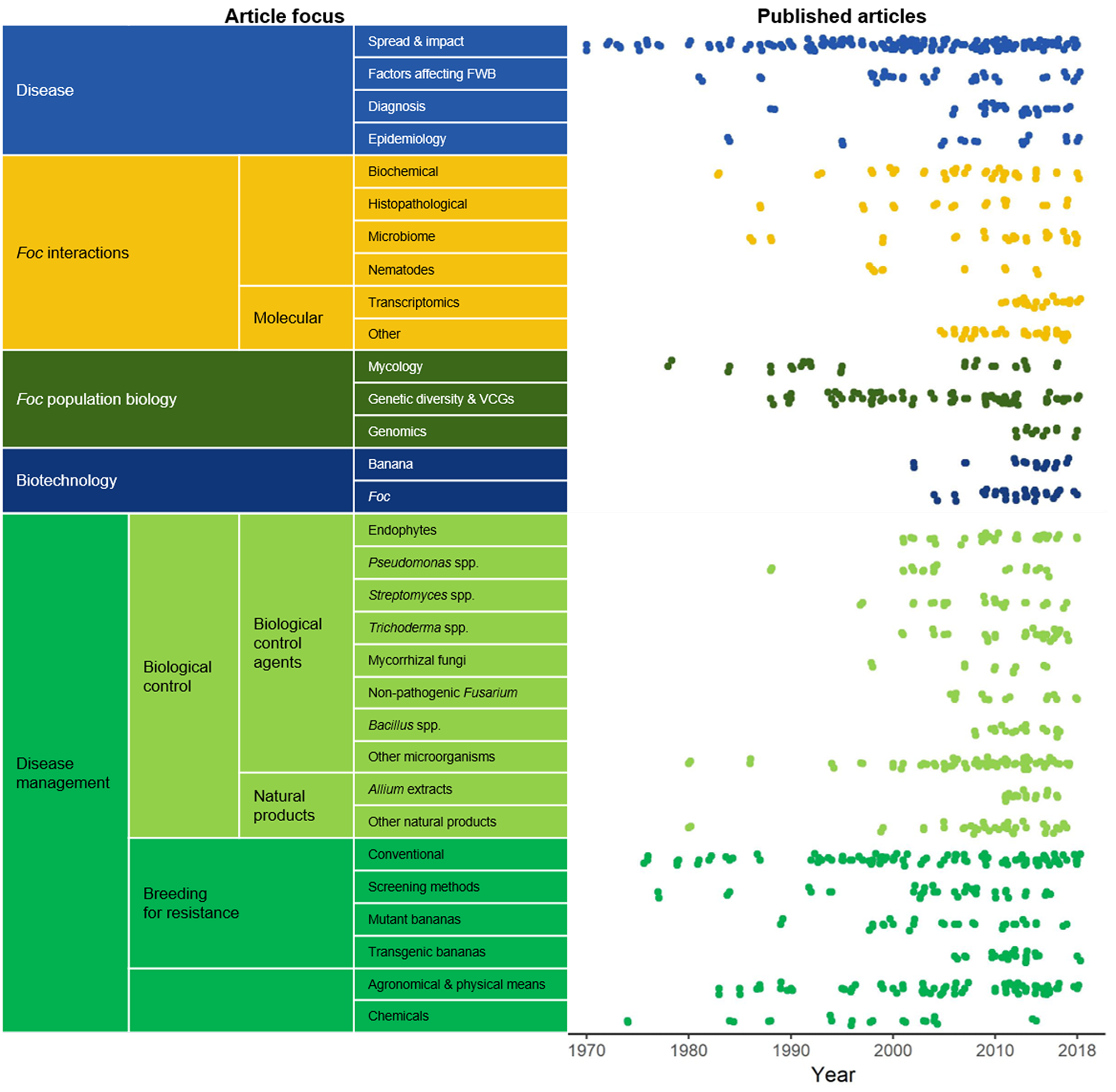

Supplement: Supplemental Figure 1 — Publication timeline of scientific articles dealing with Fusarium wilt of banana. Articles were retrieved from the CAB Direct database (1970–2018) by searching the keywords “Fusarium cubense” or “Panama disease” in the title and abstract (735 articles). Foc: Fusarium oxysporum f. sp. cubense; FWB: Fusarium wilt of banana. [file Image_1.TIF]

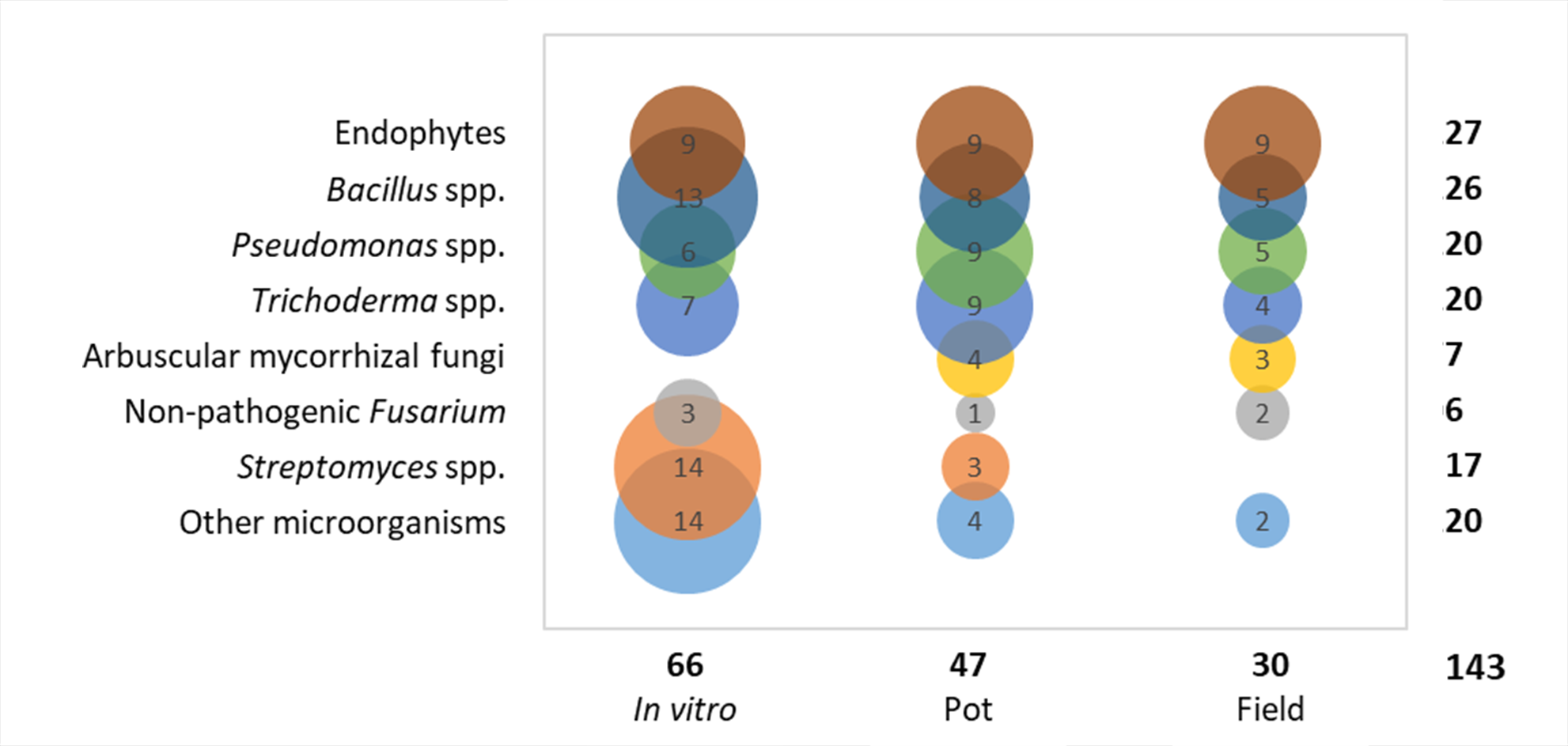

Supplement: Supplemental Figure 2 — Biological control agents studied for the control of Fusarium oxysporum f. sp. cubense. The numbers indicate the scientific articles retrieved from CAB Direct database (1970–2018) by searching the keywords “Fusarium cubense” or “Panama disease” in the title and abstract. [file Image_2.TIF]
