# Supplementary material for: Biological Control Agents Against Fusarium Wilt of Banana
Source: Front Microbiol. 2019 Apr 5;10:616. doi: 10.3389/fmicb.2019.00616 (PMC6459961; doi:10.3389/fmicb.2019.00616)
Supplement: Supplemental Table 1 — List of in vitro experiments conducted against Fusarium oxysporum f. sp. cubense (Foc) using beneficial microorganisms. [file Table_1.DOCX]

**Supplemental Table 1.** List of *in vitro* experiments conducted against *Fusarium oxysporum* f. sp. *cubense* (*Foc*) using beneficial microorganisms.

| **Source of isolation** | **Screened isolates** | **Effective strains** | | ***Foc* race** | **Assay method** | | | **Relevant remarks** | | | **Reference** | |
| --- | --- | --- | --- | --- | --- | --- | --- | --- | --- | --- | --- | --- |
| ***Endophytes*** |  |  | |  |  | | |  | | |  | |
| *Capsicum frutescens* | 108 | 2 *Bacillus subtilis* strains | |  |  | | | Bacterial population in the roots, stems, and fruits were higher in *C. frutescens* from non-irrigated than irrigated fields | | | He et al. (2002) | |
| Banana corm and root tissue | *Fusarium oxysporum* |  | |  | *In vitro* plants | | | No disease reduction | | | Niere et al. (2004) | |
| *Musa acuminata* plantations in Panyu town, Guangzhou, South China | 240 | 90% *Streptomyces* spp. | |  | Dual culture | | | More streptomycetes in healthy than wilting roots but no difference between antagonistic strains from healthy and wilting leaves | | | Cao et al. (2004) | |
| Stem and leaf tissues from various plant species |  |  | | 4 | Antagonistic activity assay | | | Plants from rural areas provided a higher recovery rate of antagonistic endophytes | | | Ting et al. (2009b) | |
| Stem tissues of *Musa* spp., grass weeds, *Mimosa pudica* and  *Allamanda* spp. | BTF05, BTF07, BTF08, BTF15, BTF21, WAA03, WAA02, MIF01, ALF01 | BTF05, BTF07, BTF08, BTF15, BTF21, WAA03, WAA02, MIF01 | | 4 | Dual culture | | | The number of volatile metabolites produced by a single strain was not associated to the biocontrol potential | | | Ting et al. (2010) | |
| Different host species | 6 endobacteria | *Herbaspirillum* spp. LCB01 and AVA02 | | 4 |  | | | Volatile organic compounds | | | Ting et al. (2011) | |
|  |  | *Trichoderma atroviride* ENDO1, 2, 3 | | 2/4 | *In vitro* plants | | | No significant differences in disease incidence but significant differences in internal symptoms | | | Ortiz and Pocasangre (2012) | |
| 5 *Musa* spp. varieties from the Amazonas state, Brazil | 122 endophytic bacteria | 4 (*B. amyloliquefacens, B. subtilis, B. thuringensis*) | |  | Dual culture | | |  | | | Souza et al. (2014) | |
| Suckers of banana cv. Grand Naine from the CAR-Indian Institute of Horticultural Research (IIHR), Bangalore, India | 47 (Actinobacteria, α- and γ-Proteobacteria, spore-forming and -nonforming Firmicutes) | 1/47 (*Pseudomonas aeruginosa* GNS.13.2a) | |  | Dual culture | | | Sucker-to-sucker variation in the associated endophytic bacteria | | | Sekhar and Pious (2015) | |
| ***Trichoderma* spp.** |  |  | |  |  | | |  | | |  | |
|  |  | *Trichoderma* spp | |  | Dual culture | | |  | | | Bernal et al. (2001) | |
|  |  | *Trichoderma* sp. strain G2 | |  | Solid and liquid culture | | | Strong inhibition of the *Foc* growth | | | Zhong et al. (2009) | |
| Experimental field of banana at Rajendra Agricultural University, Pusa, Samastipur, Bihar |  | *T. viride* | |  | Poison food and dual culture | | | Combination with carbendazim | | | Anita et al. (2014) | |
| Agricultural fields, Ecuador | 15 isolates (*T. harzianum, T. asperellum, T. virens* and *T. reesei*) | *T. harzianum* strains | |  | Dual culture | | | Antagonism against important pathogens of banana and cacao | | | Galarza et al. (2015a) | |
|  |  | *T. harzianum* | |  | Molecular analyses | | | *ThSNF1* gene encoding protein kinase | | | Galarza et al. (2015b) | |
|  |  | *T. guizhouense* NJAU 4742 (*T. harzianum* clade) | |  | Molecular analyses | | | Neutral metallopeptidase NMP1 | | | Zhang et al. (2016) | |
| Soils of Malappuram and Idukky districts of Kerala state, India |  | *T. erinaceum* Tr 9, *T. asperellum* Tr 43 | |  | Protoplasmic fusion | | | The fusant F2 exhibited 100% growth inhibition of *Pythium aphanidermatum*, *Pytophthora capsic*i and *Fusarium oxysporum*, and a 51 and 67% of *Rhizoctonia solani* and *Sclerotium rolfsii*, respectively | | | Hima et al. (2016) | |
| ***Pseudomonas* spp.** |  |  | |  |  | | |  | | |  | |
| Plant rhizosphere |  | *P. fluorescens* biotypes C and G | |  |  | | | Antagonism against several plant pathogens | | | Sakthivel et al. (1986) | |
| Rhizosphere, Terong, Perak, Malaysia | 42 | *P. fluorescens* pa4 | |  | Dual culture, culture filtrate | | | Evaluation and effectiveness of BABA and PF on *Foc* growth | | | Aeshah Mhana et al. (2011) | |
|  |  | *Pseudomonas* sp. KB2 | | 4 | Dual culture | | | Abundance of fluorescent pseudomonads, heat tolerant, and chitinolytic bacteria in the banana rhizosphere | | | Nawangsih and Purba (2013) | |
| Marine water from the Gulf of Khambhat, Gujarat |  | *P. aeruginosa* BG | |  |  | | | Growth promotion, biocontrol abilities, urease activity | | | Goswami et al. (2015) | |
| Rhizosphere from a suppressive soil | *B. subtilis*, *B. amyloliquefaciens*, *P. otitidis and P. choloeaphtis* | *P. otitidis* | | 4 |  | | | Plant growth promotion induced by fermentation broth | | | Zhang et al. (2015) | |
| ***Bacillus* spp.** |  |  | |  |  | | |  | | |  | |
|  |  | *B. subtilis* TSA3 and *B. pumilus* CH4 | | 4 | Dual culture | | | Abundance of fluorescent pseudomonads, heat tolerant, and chitinolytic bacteria in the banana rhizosphere | | | Nawangsih and Purba (2013) | |
|  |  | *Bacillus* spp. T2WF and W10 | |  |  | | | Antagonistic activity against different pathogens | | | Yang et al. (2007) | |
| Rhizosphere of healthy banana plants | 74 | *B. subtilis* S-1 | |  | Dual culture | | |  | | | Sun et al. (2008) | |
|  |  | *B. subtilis* XW-2 | |  | Dual culture, culture filtrate | | | High effectiveness of culture filtrate | | | Sun et al. (2010a) | |
| Banana plants |  | *B. subtilis* EBT1 | | 4 | Culture filtrate | | | Effect on buds multiplication and regenerated plantlets growth of ‘Taijiao 2’ banana (AAA) | | | Yang et al. (2010) | |
| Different agroclimatic conditions | 34 *B. thuringensis* | 50E and 48F | |  |  | | | Fungitoxicity and chitin-degrading activity | | | Usharani and Gowda (2011) | |
| Soil around the cultured banana root | 87 | *Bacillus* spp. 255, D5, F2, ZK11, 214, 215 and 252 | |  | Dual culture | | | Significant inhibition of *Foc*, and hyphae distortion | | | Zhou and You (2011) | |
|  |  | *B. amyloliquefaciens* NJN-6 | |  |  | | | Production of bacillomycin- and macrolactin-type antibiotics | | | Yuan et al. (2012a) | |
| China General Microbiology Culture Collection Center |  | *B. amyloliquefaciens* NJN-6 | |  | Divided plates | | | 11 out of 36 volatile compounds completely inhibited *Foc* growth | | | Yuan et al. (2012b) | |
| Fermentation cake fertilizer liquid | 29 | *B. methylotrophicus* BM-24 | | 1/4 | Dual culture | | |  | | | Huang et al. (2013) | |
| Banana rhizosphere |  | *B. subtilis* B25 | |  | Dual culture | | | Hypothetical antifungal protein | | | Tan et al. (2013) | |
|  |  | *B. subtilis* B25 | |  | Cell-free fermentation broth | | | Unknown antifungal protein | | | Yu et al. (2016) | |
|  |  | *B. subtilis* strain HJX1 | |  | Fermentation broth | | | Crude antifungal protein | | | Zhao et al. (2016) | |
| **Non-pathogenic *Fusarium* *oxysporum*** | | | |  |  | | |  | | |  | |
| PD‐suppressive soil in Kiepersol (Africa) | More than 100 |  | |  | Culture, morphological and molecular identification | | | Genetic diversity of non-pathogenic isolates of *F. oxysporum* | | | Nel et al. (2006) | |
| Banana plants cv. Robusta(AAA), Malbhog (AAB), Alpan (AAB) and Kothia (ABB) |  | *F. oxysporum* | |  | Morphological identification., pathogenicity test | | | Endophytic association of non-pathogenic *Fusarium* | | | Nita and Harsh (2015) | |
| Soil samples, different graminoids, pseudostem and roots of banana varieties Ouro, Prata and Maçã from farms in Pedra Dourada and Araponga, Brazil | 71 | *F. oxysporum* | |  | Morphological and molecular identification. Pathogenicity test | | | Horizontal transfer of genes | | | Deltour et al. (2018) | |
| ***Streptomyces* spp. and other actinomycetes** | | | |  |  | | |  | | |  | |
| Healthy and infested soil in three places, Hainan Province, China |  | *S. polychromogenes* T3-G-59 | | 4 | Dual culture | | |  | | | Zhou et al. (2016) | |
|  |  | *S. griseus* St 4 | | TR4 | Soil assay | | | Formulated cells or cell-free extracts vs. *Foc* soil inoculum | | | Zacky and Ting (2013), Zacky and Ting (2015) | |
| Botanical garden, China | 89 | *S. aureoverticillatus* HN6 | | 4 | Dual culture | | | Methanol extracts effective against 8 pathogens with EC<0.08 mg mL^-1^ | | | Wang et al. (2015) | |
| Farmland soil, Taiwan |  | *S. padanus* PMS-702 | |  | Crude culture filtrate | | | Crude culture filtrate diluted to 10 fold completely suppressed germination of *Foc* conidia | | | Shih et al. (2013) | |
| Soil, Bawangling tropical virgin  forest, Hainan, China |  | *S. albospinus* 15-4-2 | |  |  | | | Three new compounds isolated from the fermentation broth, but ineffective against *Foc*: 2-methyl-2,5,6-bornantriol, 4,4′-(3-hydroxypropane-1,1-diyl)diphenol, and 7-(4-methoxybenzyl)-4,5,6,7-tetrahydro-1,3-oxazepine-5,6-diol | | | Yu et al. (2011) | |
| China |  | *Streptomyces* sp. 702 | | 1, 4 |  | | | Fungichromin, isolated from fermentation broth, showed EC_50_ and EC_90_ of 4.7 and 3.4 µg mL^-1^, respectively, against *Foc* R1, and 21.5 and 53.1 µg mL^-1^, respectively, against *Foc* R4 | | | Wei et al. (2011) | |
| Shrimp shell, China |  | *S. roseoflavus* F-1013 | |  | Fermentation broth | | | Also effective against *Colletotrichum* sp. and *Rhizoctonia* *solani* | | | Lin and Zhang (2010) | |
| Soil samples | 398 actinomycete | 1/398 (*S. olivochromogenes.*) | |  |  | | | Antagonistic activity | | | Qiu et al. (2009) | |
|  |  | *S. noursei*  Da07210 | | 4 | Disk assay | | | Novel antibiotic 210-A, named as  (6S,8aS,9S,11S,12aR)-6-hydroxy-9,10-dimethyldecahydrobenzo[  d]azecine-2,4,12(3H)-trione | | | Wu et al. (2009b) | |
| Coastal resources | 54 | *Streptomyces* sp. g10 | | 4 | Dual culture | | | *Foc* R4 and *Rhizoctonia solani* exhibited higher resistance compared to the other fungi, such as *Pyricularia* *oryzae* and *Phytophthora* *palmivora* | | | Getha and Vikineswary (2002), Getha et al. (2004) | |
| Soil, Taiwan |  | *S. bacillaris* | |  |  | | | Effective against several fungi and bacteria; antitumor activity; production of valinomycin | | | Siu et al. (1997) | |
| Soil, Wuzhishan Primitive Area, China | 702 | *Micromonospora pattaloongensis* 210-1-61 | |  |  | | | Isolate derived from a selective isolation by using four different pretreatment methods: phenol, dry heat, SDS and ultrasonic | | | Li et al. (2010) | |
| Wheat rhizosphere soil in dry hot valley of Huili County, Sichuan province, China |  | *Streptoverticillium lavenduligriseum* GA1-2 | | 4 | Dual culture, spore germination assay | | | 36.34% mycelial growth reduction and 94.81% spore germination reduction of *Foc* R4 | | | Qi et al. (2017) | |
|  |  | Actinomycete WZ162 | |  | Fermentation broth | | | Evaluation of the effect of different conditions for the stability of the fermentation broth | | | Wu et al. (2009a) | |
| **Other microorganisms** | | |  | | |  |  | |  |  | |  |
| Orchard soil |  | Strains d4, d5, d6, B3 and p | |  | Dual culture | | |  | | | Xiao et al. (2005) | |
| Soil in banana gardens and other fruit gardens | 13 | Strains d4, d5, B3 and p | |  | Plate dual culture | | |  | | | Xiao et al. (2006) | |
| Antagonistic bacteria controlling rice blast |  |  | |  | Fermentation broth | | | Effective against several fungi | | | You et al. (2006) | |
| Soil of banana resistant to PD | 6 bacterial isolates (B05, B23, B44, B105, B133 and B146) | B05 | |  | Dual culture | | |  | | | Ma et al. (2008) | |
|  |  | *Serratia marcescens* | |  |  | | | Among the various formulations, talc powder and lignite fly ash at 40% concentration completely inhibited the growth of *Foc* | | | Sanjeevkumar et al. (2008) | |
|  |  | Bioformulations based on *Serratia marcescens* | |  |  | | | Incorporation of sucrose into bentonite showed good viability and high efficacy | | | Ting et al. (2009a) | |
| Banana rhizosphere soil | Antagonistic fungi | Strains 080409-8, 080409-13 and 080819-B2-1 | |  | Dual culture and soil assay | | |  | | | Liu et al. (2010) | |
| Waste tin mine | Antagonistic fungi | 9 strains | |  |  | | | Secondary metabolites with antifungal activity | | | Fan et al. (2011) | |
| Agricultural and forest soils in the Eastern and Northern Thailand | *Talaromyces* *austrocalifornicus*, *T. helicus* var. major, *T. indigoticus*, *T. rotundus*, T*. wortmannii*, T*. thailandiasis* and *Talaromyces* sp. 1 KUFC 3383 | *T. thailandiasis* | |  | Dual culture | | | Effective against several fungi | | | Manoch and Dethoup (2011) | |
| Banana rhizosphere |  | *Paenibacillus polymyxa* XY-10 | |  | Dual culture | | |  | | | Sun et al. (2010b) | |
|  |  | *Tsukamurella paurometabola* C-924 | |  | Dual culture | | |  | | | Marín et al. (2013) | |
| Soil in fields heavily infested by *Foc* |  | *Brevibacillus brevis* strain HIN-1 | |  | Dual culture, spore germination assay | | |  | | | Shen et al. (2013) | |
| Different habitats | 3013 | 55 | |  | Dual culture | | |  | | | Wang et al. (2014) | |
|  |  | *Eutypella* sp. ES-1 | |  |  | | | Destructive mycoparasitism | | | Sun and Hsieh (2015) | |

# References

Aeshah Mhana, M., Al-Ani, L.K.T., Lyazzat, B., and Baharuddin, S. (2011). Biological control of *Fusarium oxysporum* f. sp. *cubense* by *Pseudomonas fluorescens* and BABA *in vitro*. *World Appl. Sci. J.* 15, 189-191.

Anita, K., Rahul, K., and Harsh, K. (2014). Efficacy of fungicides and *Trichoderma viride* against *Fusarium oxysporum* f. sp. *cubense in vitro*. *The Bioscan* 9, 1355-1358.

Bernal, A., Andreu, C.M., Moya, M.M., González, M., and Fernández, O. (2001). Antagonism *in vitro* of *Trichoderma* spp. against *Fusarium oxysporum* Schlecht f. sp. *cubense* (E. F. Smith) Snyd. & Hans. *Centro Agrícola* 28, 30-32.

Cao, L., Qiu, Z., Dai, X., Tan, H., Lin, Y., and Zhou, S. (2004). Isolation of endophytic actinomycetes from roots and leaves of banana (*Musa acuminata*) plants and their activities against *Fusarium oxysporum* f. sp. *cubense*. *World J. Microbiol. Biotechnol.* 20, 501-504. doi: 10.1023/B:WIBI.0000040406.30495.48

Deltour, P., França, S.C., Heyman, L., Pereira, O.L., and Höfte, M. (2018). Comparative analysis of pathogenic and nonpathogenic *Fusarium oxysporum* populations associated with banana on a farm in Minas Gerais, Brazil. *Plant Pathol.* 67, 707-718.

Fan, H., Yang, P., Guo, Z., and Zeng, L. (2011). Screening of antifungal activity of secondary metabolites of fungi isolated from waste tin mine against *Fusarium oxysporum* f. sp. *cubense*. *Southwest China J. Agr. Sci.* 24, 604-607.

Galarza, L., Akagi, Y., Takao, K., Kim, C., Maekawa, N., Itai, A., Peralta, E., Santos, E., and Kodama, M. (2015a). Characterization of *Trichoderma* species isolated in Ecuador and their antagonistic activities against phytopathogenic fungi from Ecuador and Japan. *J. Gen. Plant Pathol.* 81, 201-210. doi: 10.1007/s10327-015-0587-x

Galarza, L., Akagi, Y., Takao, K., Peralta, E., Santos, E., and Kodama, M. (2015b). Involvement of ThSNF1 in the development and virulence of biocontrol agent *Trichoderma harzianum*. *J. Gen. Plant Pathol.* 81, 211-217. doi: 10.1007/s10327-015-0590-2

Getha, K., and Vikineswary, S. (2002). Antagonistic effects of *Streptomyces violaceusniger* strain G10 on *Fusarium oxysporum* f. sp. *cubense* race 4: indirect evidence for the role of antibiosis in the antagonistic process. *J. Ind. Microbiol. Biotechnol.* 28, 303-310. doi: 10.1038/sj.jim.7000247

Getha, K., Vikineswary, S., Wong, W.H., Seki, T., Ward, A., and Goodfellow, M. (2004). Characterization of selected isolates of indigenous *Streptomyces* species and evaluation of their antifungal activity against selected plant pathogenic fungi. *Malay. J. Sci.* 23, 37-47.

Goswami, D., Patel, K., Parmar, S., Vaghela, H., Muley, N., Dhandhukia, P., and Thakker, J.N. (2015). Elucidating multifaceted urease producing marine *Pseudomonas aeruginosa* BG as a cogent PGPR and bio-control agent. *Plant Growth Regul.* 75, 253-263. doi: 10.1007/s10725-014-9949-1

He, H., Cai, X., Hong, Y., Guan, X., and Hu, F. (2002). Selection of endophytic antifungal bacteria from *Capsicum*. *Chin. J. Biol. Control* 18, 171-175.

Hima, V.M., Beena, S., and Cherian, K.A. (2016). Protoplasmic fusion: a biotechnological tool for the enhancement of bio-efficacy of native isolates of *Trichoderma* spp. *Indian Phytopathology* 69, 649-651.

Huang, X., Chen, B., Zhou, D., Tan, X., and Zhang, X. (2013). Isolation, identification of BM-24 strains and its antifungal activity of antagonistic bacteria against *Fusarium oxysporum* f. sp. *cubense*. *Acta Phytophylacica Sinica* 40, 121-127.

Li, Z., Liao, D., Chen, H., and Zeng, H. (2010). Isolation and identification of a strain of rare actinomycetes with antagonistic activity against *Fusarium oxyporm* f. sp. *cubense*. *Genomics Appl. Biol.* 29, 303-309.

Lin, M., and Zhang, S. (2010). The antagonistic effect of shrimp fermentation liquor of *Streptomyces* F-1013 against plant pathogenic fungi. *J. Fujian Agric. Univ.* 39, 584-589.

Liu, C., Wang, G., Liang, C., Wang, J., Yang, L., Qin, H., Wu, L., and Huang, J. (2010). Comparision of antagonistic efficacy about three fungi against banana Fusarium wilt. *J. Fruit Sci.* 27, 1032-1036.

Ma, H., Yang, X., Ruan, H., Du, Y., and Chen, F. (2008). *In vitro* study of the antagonistic activity of some microorganisms against *Fusarium oxysporum* f. sp. *cubense*. *Fujian J. Agric. Sci.* 23, 251-254.

Manoch, L., and Dethoup, T. (2011). A potential use of *Talaromyces* species as biological agents against plant pathogenic fungi. *Thai J. Agric. Sci.* 44, 81-91.

Marín, M., Wong, I., García, G., Morán, R., Basulto, R., Pimentel, E., and Mena, J. (2013). *In vitro* antagonistic activity of *Tsukamurella paurometabola* C-924 against phytopathogens. *Revista de Protección Vegetal* 28, 132-137.

Nawangsih, A.A., and Purba, F. (2013). Isolation of fluorescent pseudomonads, heat tolerant and chitinolytic bacteria in banana rhizosphere with antagonistic activities against *Fusarium oxysporum* f. sp. *cubense in vitro* and molecular identification of selected isolates. *J. Int. Soc. Southeast Asian Agr. Sci.* 19, 30-40.

Nel, B., Steinberg, C., Labuschagne, N., and Viljoen, A. (2006). Isolation and characterization of nonpathogenic *Fusarium oxysporum* isolates from the rhizosphere of healthy banana plants. *Plant Pathol.* 55, 207-216. doi: 10.1111/j.1365-3059.2006.01343.x

Niere, B., Gold, C.S., and Coyne, D. (2004). Can fungal endophytes control soilborne pests in banana? *Bulletin OILB/SROP* 27, 203-209.

Nita, K., and Harsh, K. (2015). Association of nonpathogenic *Fusarium oxysporum* species with cultured shoot apices of banana (*Musa acuminata*) cultivars. *The Bioscan* 10, 629-633.

Ortiz, R., and Pocasangre, L.E. (2012). Biological control of Panama disease (*Fusarium oxysporum* f. sp. *cubense*) using endophytic fungi. *Tierra Tropical: Sostenibilidad, Ambiente y Sociedad* 8, 221-228.

Qi, D., Zou, L., Zhou, D., Feng, R., Gao, Z., and Zhang, X. (2017). Isolation, identification of strain GA1-2 and its antifungal activity against *Fusarium oxysporum* f. sp. *cubense*. *J. Plant Prot.* 44, 809-816.

Qiu, W., Huang, H., Ye, J., and Bao, S. (2009). Screening of actinomycetes against *Fusarium oxysporum* f. sp. *cubense* and identification of strain DA07408. *Res. Agr. Modernization* 30, 126-128.

Sakthivel, N., Sivamani, E., Unnamalai, N., and Gnanamanickam, S.S. (1986). Plant growth-promoting rhizobacteria in enhancing plant growth and suppressing plant pathogens. *Curr. Sci. India* 55, 22-25.

Sanjeevkumar, K., Eswaran, A., and Muthukumar, A. (2008). Survival of *Serratia marcescens* in different carrier materials and mycelial dry weight of *Fusarium oxysporum* f. sp. *cubense*. *Plant Arch.* 8, 345-346.

Sekhar, A.C., and Pious, T. (2015). Isolation and identification of shoot-tip associated endophytic bacteria from banana cv. Grand Naine and testing for antagonistic activity against *Fusarium oxysporum* f. sp. *cubense*. *Am. J. Plant Sci.* 6, 943-954. doi: 10.4236/ajps.2015.67101

Shen, L., Xiong, G., Dong, L., Kong, R., Guo, T., and Zhang, S. (2013). Identification of a strain HN-1 against banana wilt disease and determination of its antagonism. *Plant Dis. Pests* 4, 12-16.

Shih, H.D., Chung, W.C., Huang, H.C., Tseng, M., and Huang, J.W. (2013). Identification for *Streptomyces padanus* strain PMS-702 as a biopesticide agent. *Plant Pathol. Bull.* 22, 145-158.

Siu, M., Wang, S., Lu, S., and Hsu, Y. (1997). Isolation of valinomycin producing *Streptomyces* from Taiwan soil. *J. Chin. Agric. Chem. Soc.* 35, 243-251.

Souza, A., Cruz, J.C., Sousa, N.R., Procópio, A.R.L., and Silva, G.F. (2014). Endophytic bacteria from banana cultivars and their antifungal activity. *Genet. Mol. Res.* 13, 8661-8670. doi: 10.4238/2014.October.27.6

Sun, J., Wang, Y., Li, W., and Peng, M. (2010a). Screening and identification of chitinase-producing bacterium and its antagonistic activity against *Fusarium oxysporum* f. sp. *cubense*. *J. Fruit Sci.* 27, 427-430.

Sun, J., Wang, Y., Zhao, P., and Peng, M. (2010b). Isolation, identification and antagonistic acitivity of biocontrol bacterium against *Fusarium oxysporum* f. sp. *cubense*. *Chin. J. Biol. Control* 26, 347-351.

Sun, T., and Hsieh, F. (2015). First record of *Eutypella* sp. as a mycoparasite on *Fusarium oxysporum* f. sp. *cubense*. *Plant Prot. Bull. Taiwan* 57, 25-30.

Sun, Z., Ji, C., Li, Y., and Wang, Z. (2008). Antagonistic rhizobacteria strain *Bacillus subtilis* S-1 against banana Fusarium wilt. *Chin. J. Biol. Control* 24, 143-147.

Tan, Z., Lin, B., and Zhang, R. (2013). A novel antifungal protein of *Bacillus subtilis* B25. *SpringerPlus* 2, 543. doi: 10.1186/2193-1801-2-543

Ting, A.S.Y., Fang, M.T., and Tee, C.S. (2009a). Assessment on the effect of formulative materials on the viability and efficacy of *Serratia marcescens* - a biocontrol agent against *Fusarium oxysporum* f. sp. *cubense* race 4. *Am. J. Agr. Biol. Sci.* 4, 283-288. doi: 10.3844/ajabssp.2009.283.288

Ting, A.S.Y., Mah, S.W., and Tee, C.S. (2009b). Prevalence of endophytes antagonistic towards *Fusarium oxysporum* f. sp. *cubense* race 4 in various plants. *Am.-Eurasian J. Sustain. Agr.* 3, 399-406.

Ting, A.S.Y., Mah, S.W., and Tee, C.S. (2010). Identification of volatile metabolites from fungal endophytes with biocontrol potential towards *Fusarium oxysporum* f. sp. *cubense* race 4. *Am. J. Agr. Biol. Sci.* 5, 177-182. doi: 10.3844/ajabssp.2010.177.182

Ting, S., Mah, S., and Tee, C. (2011). Detection of potential volatile inhibitory compounds produced by endobacteria with biocontrol properties towards *Fusarium oxysporum* f. sp. *cubense* race 4. *World J. Microbiol. Biotechnol.* 27, 229-235. doi: 10.1007/s11274-010-0447-y

Usharani, T.R., and Gowda, T.K.S. (2011). Cloning of chitinase gene from *Bacillus thuringiensis*. *Indian J. Biotechnol.* 10, 264-269. doi: 10.2323/jgam.43.341

Wang, F., Lyu, S., Liu, W., Zeng, L., Du, C., Zhou, J., Han, X., and Liu, J. (2014). Screening of antagonistic bacteria against *Fusarium oxysporum* f. sp. *cubense* and analysis of the substances associated with biological control. *Acta Agric. Univ. Jiangxiensis* 36, 1264-1269.

Wang, L., Xing, M., Di, R., and Luo, Y. (2015). Isolation, identification and antifungal activities of *Streptomyces aureoverticillatus* HN6. *J. Plant Pathol. Microbiol.* 6, 281. doi: 10.4172/2157-7471.1000281

Wei, S., Zhang, Z., Tu, X., He, J., and Tu, G. (2011). Studies on the isolation, identification and activity of anti-*Fusarium oxysporum* secondary metabolites produced by *Streptomyces* sp. 702. *Acta Agric. Univ. Jiangxiensis* 33, 982-986.

Wu, Q., Zeng, H., and Gong, S. (2009a). Stability of fermentation broth of actinomycete strain WZ162 resistance to *Fusarium oxysporum* f. sp. *cubense* of banana. *Guangxi Agr. Sci.* 40, 366-369.

Wu, X., Huang, H., Chen, G., Sun, Q., Peng, J., Zhu, J., and Bao, S. (2009b). A novel antibiotic produced by *Streptomyces noursei* Da07210. *Antonie Van Leeuwenhoek* 96, 109-112. doi: 10.1007/s10482-009-9333-8

Xiao, A., Li, G., You, C., and Huang, Y. (2005). Inhibition of five antagonistic bacteria to *Fusarium oxysporum* f. sp. *cubense*. *Acta Agric. Univ. Jiangxiensis* 27, 572-575.

Xiao, A., You, C., Liang Guan, P., and Huang, Y. (2006). Selection of antagonistic bacteria against *Fusarium oxysporum* f. sp.*cubense* and their action mechanism. *Plant Prot.* 32, 53-56.

Yang, X., Chen, F., Gan, L., Du, Y., and Ruan, H. (2010). Effect of the endophytic *Bacillus subtilis* EBT1 isolated from banana on the growth and resistance to Fusarium wilt disease in banana. *Acta Phytophylacica Sinica* 37, 300-306.

Yang, X., He, Y., Chen, F., and Ruan, H. (2007). Identification and colonization of antagonists T2WF and W10 against *Fusarium oxysporum* f. sp. *cubense*, pathogen of banana wilt disease. *Chin. J. Biol. Control* 23, 73-77.

You, C., Xiao, A., Fu, Z., and Zhen, J. (2006). The inhibitive effect of antagonistic bacterium controlling rice blast on four soil-borne pathogenic fungi. *Acta Agric. Univ. Jiangxiensis* 28, 860-863, 867.

Yu, J., Zhang, R., Tan, Z., and Lin, B. (2016). Studies on antifungal activity and purification of antifungal substance from *Bacillus subtilis* B25 strain. *Genomics Appl. Biol.* 35, 629-634.

Yu, L., Dai, H., Zhao, Y., Zeng, Y., Jiang, W., Mei, W., and Zeng, H. (2011). Three new compounds from soil actinomycete *Streptomyces albospinus* 15-4-2. *J. Asian Nat. Prod. Res.* 13, 901-906. doi: 10.1080/10286020.2011.599322

Yuan, J., Li, B., Zhang, N., Waseem, R., Shen, Q., and Huang, Q. (2012a). Production of bacillomycin- and macrolactin-type antibiotics by *Bacillus amyloliquefaciens* NJN-6 for suppressing soilborne plant pathogens. *J. Agric. Food Chem.* 60, 2976-2981. doi: 10.1021/jf204868z

Yuan, J., Raza, W., Shen, Q., and Huang, Q. (2012b). Antifungal activity of *Bacillus amyloliquefaciens* NJN-6 volatile compounds against *Fusarium oxysporum* f. sp. *cubense*. *Appl. Environ. Microbiol.* 78, 5942-5944. doi: 10.1128/AEM.01357-12

Zacky, F.A., and Ting, A.S.Y. (2013). Investigating the bioactivity of cells and cell-free extracts of *Streptomyces griseus* towards *Fusarium oxysporum* f. sp. *cubense* race 4. *Biol. Control* 66, 204-208. doi: 10.1016/j.biocontrol.2013.06.001

Zacky, F.A., and Ting, S. (2015). Biocontrol of *Fusarium oxysporum* f. sp. *cubense* tropical race 4 by formulated cells and cell-free extracts of *Streptomyces griseus* in sterile soil environment. *Biocontrol Sci. Technol.* 25, 685-696. doi: 10.1080/09583157.2015.1007921

Zhang, H., Song, Y., Lu, S., Guo, J., and Zeng, R. (2015). The antifungal activity and crop growth stimulation of growth-promoting rhizobacteria from banana rhizosphere soil. *J. S. China Agric. Univ.* 36, 65-70.

Zhang, J., Akcapinar, G.B., Atanasova, L., Rahimi, M.J., Przylucka, A., Yang, D., Kubicek, C.P., Zhang, R., Shen, Q., and Druzhinina, I.S. (2016). The neutral metallopeptidase NMP1 of *Trichoderma guizhouense* is required for mycotrophy and self-defence. *Environ. Microbiol.* 18, 580-597. doi: 10.1111/1462-2920.12966

Zhao, G., Qiu, Y., Zhang, Y., and Xiong, G. (2016). Preliminary study on production conditions and action mechanism of antifungal protein from strain HJX1. *Agric. Biotechnol.* 5, 46-48.

Zhong, X., Liang, M., Zhen, X., Lai, J., and Lai, X. (2009). Study on the inhibition of *Trichoderma* sp. against *Fusarium oxysporum* f. sp. *cubense* in banana. *J. Fruit Sci.* 26, 186-189.

Zhou, D., Jing, T., Zhang, X., Qi, D., Chen, Y., and Wang, F. (2016). Screening and antibacterial activity of antagonistic bacteria against banana Fusarium wilt disease. *Acta Phytophylacica Sinica* 43, 913-921.

Zhou, J., and You, C. (2011). Screening and identification of antagonistic bacteria against *Fusarium oxysporum* f. sp. *cubense* race 4. *J. Fruit Sci.* 28, 278-283.
